# A coordination cage hosting ultrafine and highly catalytically active gold nanoparticles†

**DOI:** 10.1039/d1sc05407d

**Published:** 2021-12-07

**Authors:** Xinxin Hang, Shentang Wang, Huan Pang, Qiang Xu

**Affiliations:** School of Chemistry and Chemical Engineering, Institute for Innovative Materials and Energy, Yangzhou University Yangzhou 225002 P. R. China huanpangchem@hotmail.com qxuchem@yzu.edu.cn; School of Chemistry and Chemical Engineering, Chongqing Key Laboratory of Soft-Matter Material Chemistry and Function Manufacturing, Southwest University Chongqing 400715 P. R. China wsht212@swu.edu.cn; Department of Materials Science and Engineering, SUSTech Academy for Advanced Interdisciplinary Studies, Southern University of Science and Technology (SUSTech) Shenzhen 518055 P. R. China; Institute for Integrated Cell-Material Sciences (iCeMS), Kyoto University Yoshida, Sakyo-ku Kyoto 606-8501 Japan xu.qiang@icems.kyoto-u.ac.jp

## Abstract

Ultrafine metal nanoparticles (MNPs) with size <2 nm are of great interest due to their superior catalytic capabilities. Herein, we report the size-controlled synthesis of gold nanoparticles (Au NPs) by using a thiacalixarene-based coordination cage CIAC-108 as a confined host or stabilizer. The Au NPs encapsulated within the cavity of CIAC-108 (Au@CIAC-108) show smaller size (∼1.3 nm) than the ones (∼4.7 nm) anchored on the surface of CIAC-108 (Au/CIAC-108). The cage-embedded Au NPs can be used as a homogeneous catalyst in a mixture of methanol and dichloromethane while as a heterogeneous catalyst in methanol. The homogeneous catalyst Au@CIAC-108-homo exhibits significantly enhanced catalytic activities toward nitroarene reduction and organic dye decomposition, as compared with its larger counterpart Au/CIAC-108-homo and its heterogeneous counterpart Au@CIAC-108-hetero. More importantly, the as-prepared Au@CIAC-108-homo possesses remarkable stability and durability.

## Abstract 2

The size-controlled synthesis of Au NPs was achieved by using a coordination cage CIAC-108 as a support. The Au NPs encapsulated within the cavity of CIAC-108 show smaller size (∼1.3 nm) than the ones (∼4.7 nm) anchored on the surface of CIAC-108.

## Graphical abstract

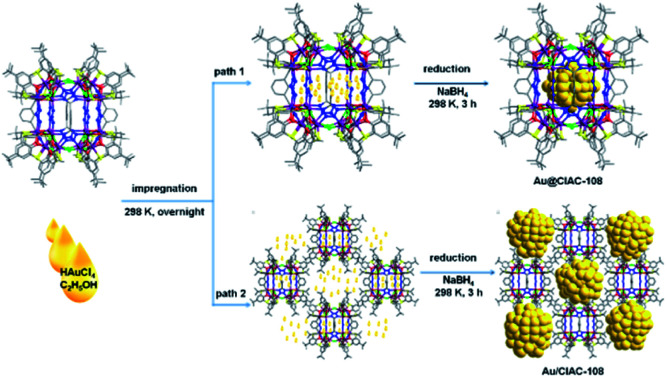

## Introduction

Ultrafine and well-dispersed metal nanoparticles (MNPs) have attracted extensive research interest in catalysis owing to their excellent catalytic capabilities and advanced applications.^1–4^ Metal particles with a size of a few nanometers or less exhibit fascinating properties differing significantly from their bulk counterparts.^5–8^ In particular, MNPs with diameters ranging from subnanometer to ∼2 nm, possess well-defined electronic structures and intriguing molecular-like properties because electron energy quantization occurs in this ultrasmall size regime.^9–11^ Unfortunately, such tiny MNPs with high surface-energy are thermodynamically unstable and prone to agglomerate and coalescence during catalysis reactions, resulting in a dramatic decrease in catalytic activity, thus limiting their practical applications.^12–14^ To address this issue, encapsulating metal species into porous materials, such as metal–organic frameworks,^15–20^ covalent–organic frameworks,^21–23^ and organic cages,^24–33^ has been demonstrated to be a powerful strategy to engineer the controlled synthesis and stabilization of ultrafine MNPs. However, the synthesis of such ultrafine MNPs with a narrow size distribution and high stability is still a big challenge.

Coordination cages with large internal voids^34–40^ have aroused enormous attention owing to their aesthetically elegant architectures^41–45^ and widespread applications in gas separation and storage,^46,47^ enantioseparation,^48^ and catalysis.^49–51^ Very recently, coordination cages have emerged as an effective platform for confined synthesis of ultrafine MNPs due to their unique advantages. Coordination cages with a confined cavity environment are expected to physically isolate MNPs and inhibit their aggregation, and thus improve their catalytic activity and stability.^52,53^ In addition, the tunable cavity offers great opportunity for coordination cages to tune the size^54–56^ and morphology^57,58^ of encapsulated MNPs. Furthermore, when encapsulated inside soluble coordination cages, MNPs are endowed with high dispersibility in solution.^59^ Although a few successful examples have been reported in this field,^52–59^ the encapsulation of tiny MNPs (especially diameter <2 nm) within coordination cages remains challenging.

Herein, we report the size-, and location-controlled synthesis of Au NPs using a thiacalixarene-based coordination cage, CIAC-108, as a support. When a low concentration of the Au precursor is employed, ultrafine Au NPs with small size (∼1.3 nm) encapsulated in the cavity of CIAC-108 (Au@CIAC-108) was achieved. In contrast, large Au NPs (∼4.7 nm) occurred on the cage periphery (Au/CIAC-108) if a high-concentration of the Au precursor is chosen. The as-prepared Au NPs can serve as a homogeneous catalyst in a mixture of methanol and dichloromethane while as a heterogeneous catalyst in methanol solution. More intriguingly, the homogeneous catalyst Au@CIAC-108-homo exhibits remarkable catalytic activity for various chemical transformations, along with excellent stability and durability that there is no significant decline in catalytic performance over 6 cycles.

## Results and discussion

### Synthesis and characterization of CIAC-108

CIAC-108 is synthesized according to a modified literature method.^60^ Fourier transform infrared (FT-IR) spectra of the product are all consistent with those previously reported (Fig. S1†), confirming the successful formation of CIAC-108. Power X-ray diffraction (PXRD) measurements demonstrate that desolvated CIAC-108 shows weak diffraction peaks in the range 5–50° in which the crystal X-ray diffraction data are collected owing to the loss of crystallinity upon solvent removal (Fig. S2†). Interestingly, strong diffraction peaks in the region 3–5° of desolvated CIAC-108 are observed (Fig. S3†). Based on the crystal structure of CIAC-108, the inner cavity is 12.2 × 12.2 × 12.5 Å^3^, and the size of the nanocage is 24.0 × 23.5 × 25.8 Å^3^. CIAC-108 is not soluble in some polar solvents, such as water, methanol, and alcohol, but can dissolve in a methanol–dichloromethane mixture in a certain volume ratio or dichloromethane.

### Synthesis and characterization of coordination cage-embedded Au NPs

**Fig. 1:**
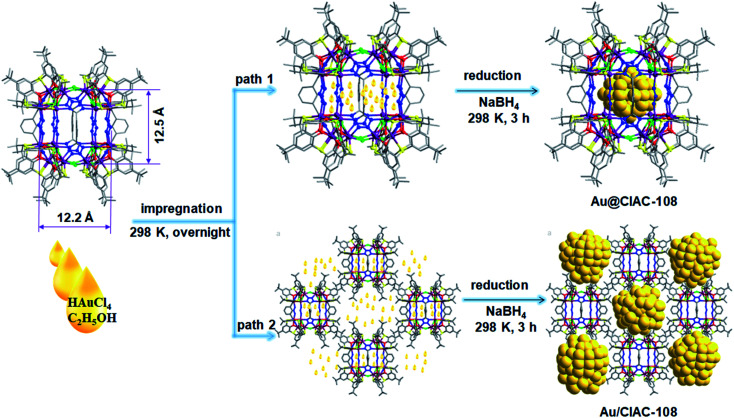
Schematic representation of the synthesis of Au@CIAC-108 and Au/CIAC-108.

**Fig. 2:**
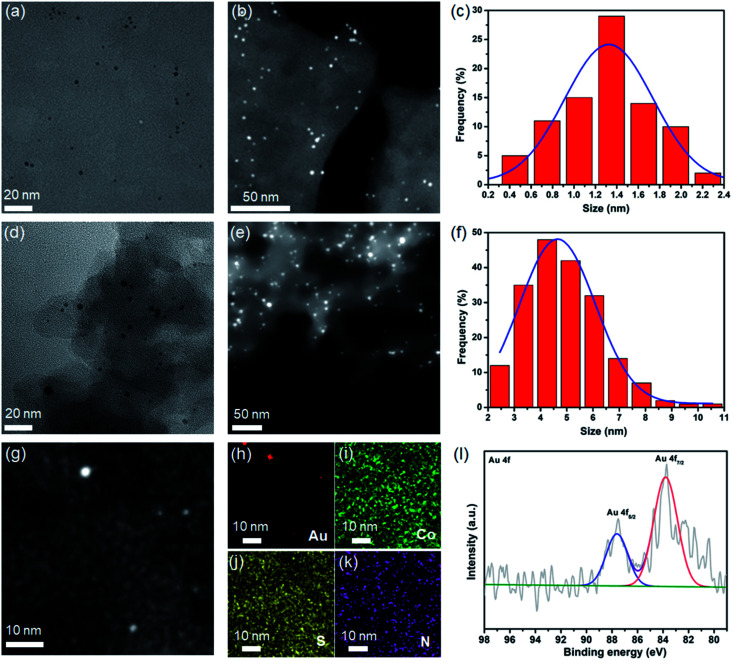
(a and d) TEM images; (b and e) HAADF-STEM images; (c and f) the histogram of particle-size distributions of Au@CIAC-108 and Au/CIAC-108, respectively. (g–k) EDS mapping images of Au@CIAC-108. (l) XPS spectrum of Au NPs.

Coordination cages with well-defined nanometer-sized cavities make them a potential template for the controlled growth of ultrafine MNPs. The thiacalixarene-based coordination cage, CIAC-108, was selected as a support for the fabrication of encapsulated Au NPs owing to the following unique features: (1) high stability and good solubility in certain solvents; (2) cage cavity with pore windows large enough for metal precursor diffusion; and most importantly, (3) the interior of the hydrophobic cavity decorated with abundant nitrogen atoms that would facilitate the metal deposition, and the nucleation and growth of MNPs. The cage-supported Au NPs were synthesized by a wet-chemistry approach. As shown by path 1 in Fig. 1, an ethanol solution (20 mL) of the CIAC-108 sample (80 mg) was treated with 5 mL of ethanol solution with HAuCl_4_ (2 mg mL^−1^, 240 μL). After vigorously stirring for 3 h, the as-prepared mixture was subsequently reduced with an ethanol solution of NaBH_4_ (2.7 M, 0.25 mL). The red turbid solution immediately turned black without no precipitation, indicating the efficient reduction of the Au^3+^ ions to Au^0^, and further stabilization of the resulting Au NPs by CIAC-108. The resulting Au@CIAC-108 was isolated by centrifugation, washed with methanol and dried in air. The PXRD pattern of the as-obtained Au@CIAC-108 is consistent with that of desolvated CIAC-108 (Fig. S3†), indicating that the structural integrity of CIAC-108 is maintained well even after strong treatment with NaBH_4_. Meanwhile, no diffraction peaks of Au NPs are detected, suggesting the successful encapsulation of ultrafine Au NPs inside the CIAC-108 cavity. The presence of all the characteristics peaks of CIAC-108 in the FT-IR spectrum of Au@CIAC-108 further confirms that the Au NP loading occurred without disturbing the CIAC-108 architecture (Fig. S1†). The transmission electron microscopy (TEM) image (Fig. 2a) shows that ultrafine Au NPs are well-dispersed and have an average diameter of 1.3 nm with a narrow size distribution of 0.4–2.1 nm (Fig. 2c), which is consistent with the estimated pore size of the cage cavity, indicating that the majority of the Au NPs are well encapsulated within CIAC-108. The formation of Au@CIAC-108 is further confirmed from the high-angle annular dark-field scanning transmission electron microscopy (HAADF-STEM) image (Fig. 2b), which reveals that Au NPs are uniformly distributed inside the CIAC-108 without any agglomeration. Energy-dispersive X-ray spectroscopy (EDS) mapping analysis revealed that the component elements Au, Co, S, and N are all evenly distributed in AuNPs@CIAC-108 (Fig. 2g–k), further confirming that the Au NPs are homogeneously distributed inside the cavity of CIAC-108. X-ray photoelectron spectroscopy (XPS) analysis was performed to identify the chemical states of the Au element of Au@CIAC-108. The peaks centered at 87.6 and 83.9 eV correspond to two distinct spin–orbit pairs of Au 4f_5/2_ and 4f_7/2_, indicating the dominance of metallic Au species in Au@CIAC-108 (Fig. 2l).^29,32^ When a high-concentration HAuCl_4_ solution (2 mg mL^−1^, 350 μL) is employed, the precursors would be redissolved and diffuse out of the cage cavity, affording larger Au NPs embedded on the outer surface of CIAC-108 (path 2 in Fig. 1). TEM and HAADF-STEM analyses showed that uniformly dispersed Au NPs (Fig. 2d and e) with an average size of 4.7 nm were formed (Fig. 2f), which is larger than the estimated cage window size and internal cavity size, suggesting that most of the particles are embedded on the external surface of CIAC-108, where Au NPs could be stabilized by multiple nitrogen atoms and sulfur atoms (Au/CIAC-108). These results demonstrated that the size and location of Au NPs can be achieved by the precise control of the concentration of precursors.

To clarify the critical role of CIAC-108 in the size-controlled growth of Au NPs, a control experiment in the absence of CIAC-108, in which only *p-tert*-butylthiacalix[4]arene (H_4_TC4A), 1,3-dicyanobenzene (IPN), and sodium azide (NaN_3_) were used as the stabilizing ligands, was performed. The TEM image showed the formation of structure-less agglomerates and black precipitates (denoted as the Au/(H_4_TC4A–IPN–NaN_3_ mixture), Fig. S4†). This observation indicated that in the absence of CIAC-108, there is lack of control on the deposition sites of MNPs, and thus MNPs of random size are formed.

The as-prepared Au@CIAC-108 and Au/CIAC-108 are insoluble but readily dispersed in strong polar solvents, such as water, methanol, and alcohol and can be dissolved in a methanol–dichloromethane solvent mixture at a certain volume ratio and give clear solutions without any precipitation, making them promising candidates as homogeneous or heterogeneous catalysts.

### Catalytic properties of cage-embedded Au NPs

**Fig. 3:**
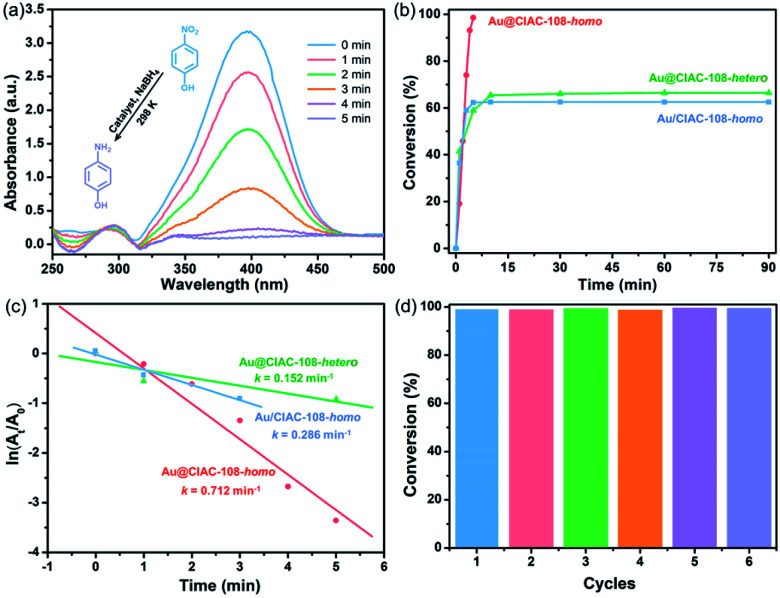
(a) Time-dependent evolution of UV-Vis spectra of the reduction of 4-NPh to 4-APh catalyzed by Au@CIAC-108-homo; (b) comparative catalytic conversion of 4-NPh by Au@CIAC-108-homo, Au/CIAC-108-homo, and Au@CIAC-108-hetero; (c) plot of ln(*A*_*t*_/*A*_0_) for the absorbance of 4-NPh at 400 nm from the spectra *versus* time for the reduction of 4-NPh catalyzed by Au@CIAC-108-homo, Au/CIAC-108-homo (in the initial 3 min), and Au@CIAC-108-hetero (in the initial 5 min) catalysts; (d) durability test for the reduction of 4-NPh over the Au@CIAC-108-homo catalyst.

It is well-known that Au NPs are chemically stable, possessing exceptional properties, and have been extensively used in various chemical transformations.^61–63^ To evaluate the catalytic activity of the cage-stabilized Au NPs, the catalytic hydrogenation of 4-nitrophenol (4-NPh) in the presence of 10 mg NaBH_4_ to form 4-aminophenol (4-APh) was first selected as a test reaction. The catalytic reaction was initiated by adding 4-NPh to a flask containing a methanol–dichloromethane mixture (volume ratio = 1 : 2), Au@CIAC-108 (Au/CIAC-108) and NaBH_4_ with vigorous shaking. It is notable that Au@CIAC-108 and Au/CIAC-108 are soluble in this solution, serving as homogenous catalysts (denoted as Au@CIAC-108-homo and Au/CIAC-108-homo, respectively). The reduction kinetics of 4-NPh to 4-APh was monitored using ultraviolet-visible (UV-Vis) absorption spectra. The characteristic peak of 4-NPh at 400 nm decreases along with the concomitant increase of the characteristic absorbance of 4-APh at ∼300 nm (Fig. 3a). The complete hydrogenation of 4-NPh to 4-APh by Au@CIAC-108-homo only needs 5.0 min (*n*(Au)/*n*(4-NPh) = 1/263) (Fig. 3b). For comparison, the Au/CIAC-108-homo counterpart with larger particle sizes exhibits lower catalytic activity with 62.5% conversion in 90 min (*n*(Au)/*n*(4-NPh) = 1/156) (Fig. 3b). The pseudo-first-order kinetic model was applied to evaluate the kinetic reaction rates of 4-NPh and the related linear correlations of ln(*A*_*t*_/*A*_0_) *versus* reaction time (*A*_*t*_ and *A*_0_ represent absorbance of the 4-NPh at the intervals and at the initial reaction stage, respectively) were obtained. The Au@CIAC-108-homo catalyst shows a remarkably high rate constant of 0.712 min^−1^ (Fig. 3c). In contrast, the estimated rate constant of the catalyst Au/CIAC-108-homo is only 0.286 min^−1^ (Fig. 3c), indicating that Au@CIAC-108-homo shows an enhanced catalytic performance. Notably the catalytic activity of Au@CIAC-108-homo is comparable to that of the previously-reported Au NP catalysts under similar conditions (Table S1†), *e.g.* Au@SiO_2_ yolk/shell nanoreactor,^64^ Au@Ag/ZIF-8,^65^ and Au@PCC-1,^32^ and is superior to that of Au(0)@TpPa-1,^23^ ZIF-8 NC@Au,^66^ and Au@MIL-100(Fe).^67^ The catalytic activity of Au@CIAC-108-homo was then examined by the hydrogenation of 4-nitroaniline (4-NAn) to form 4-phenylene diamine (4-DPN) under similar conditions. As shown in Fig. S18a,† the Au@CIAC-108-homo catalyst gave complete conversion in 1.0 min (*n*(Au)/*n*(4-NAn) = 1/693), which can be estimated by recording the change of the characteristic absorbance at 370 nm. The Au@CIAC-108-homo catalyst showed a remarkably high rate constant of 5.41 min^−1^ (Fig. S18c†), which was superior to those of reported catalysts.^27,32^ Moreover, Au@CIAC-108-homo exhibited significantly enhanced catalytic activity, high conversion and constant *k* values in the catalytic hydrogenation of other nitroarene derivatives, including 2-nitroaniline (Fig. S11†) and 3-nitroaniline (Fig. S15†) compared with its Au/CIAC-108-homo counterpart (Fig. S12 and S16†). Impressively, Au@CIAC-108-homo exhibited boosted catalytic activities toward 2-nitroaniline (Fig. S14†) and 4-nitroaniline (Fig. S21†) even when a half dosage of NaBH_4_ (5 mg) was used. Note that the constant *k* value of Au@CIAC-108-homo in the catalytic reduction of 2-nitrophenol is slightly smaller than that of Au/CIAC-108-homo which may be attributed to the high Au NP loading of Au/CIAC-108-homo (Fig. S9†). These experimental results further support the well-established fact that the catalytic activity of MNPs is heavily dependent on their size, wherein a decrease in size leads to an improvement in their catalytic activity. For comparison, the catalytic reductions of these nitroarenes without any catalyst were performed. As expected, negligible performance was observed (Fig. S22†). This observation demonstrated that the reduction of nitroarenes hardly occurred in the presence of NaBH_4_ but without the Au NP catalyst, which was confirmed in the previously-reported work.^64^ Moreover, Au/(H_4_TC4A–IPN–NaN_3_ mixture) and the CIAC-108 catalysts were investigated as catalysts for these reactions, which showed lower catalytic activity (Fig. S23 and S24†).

It has been well documented that homogeneous catalysts generally exhibit higher reaction activity than their heterogeneous counterparts because of their well-dispersed catalytic sites in solution, which facilitates the direct contact between the catalysts and reactants.^68,69^ Thus, Au@CIAC-108 serving as a heterogeneous catalyst (denoted as Au@CIAC-108-hetero) toward the reduction of 4-NPh to 4-APh is compared. The reaction was carried out under similar conditions except the mixture of methanol–dichloromethane (volume ratio = 1 : 2) is replaced by methanol solution, in which Au@CIAC-108 is insoluble that provides the heterogeneous conditions but could be well-dispersed. As shown in Fig. 3b, the hydrogenation reaction by the Au@CIAC-108-hetero catalyst can only be achieved with a yield of 66.4% after 90 min (*n*(Au)/*n*(4-NPh) = 1/40), which is much lower than that of Au@CIAC-108-homo. The estimated rate constant of the Au@CIAC-108-hetero catalyst is only 0.152 min^−1^ (Fig. 3c). The catalytic hydrogenation of other nitroarene derivatives also demonstrated that the catalytic activity of Au@CIAC-108-homo is superior to that of Au@CIAC-108-hetero (Fig. S10, S13, S17 and S20†).

**Fig. 4:**
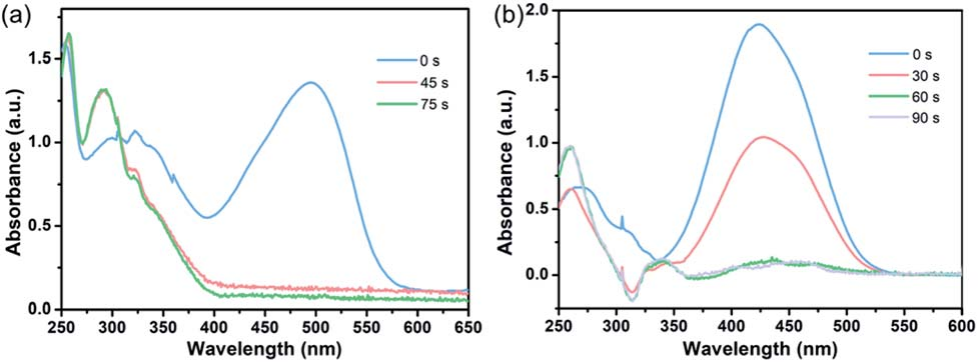
UV-Vis absorption spectra of the catalytic decomposition of Congo red (a) and methyl orange (b) in the presence of the Au@CIAC-108-homo catalyst.

To further confirm the boosting of catalytic activity by the Au@CIAC-108-homo catalyst, the catalytic decomposition of organic dyes (Congo red and methyl orange) was investigated as another representative probe reaction. The characteristic absorption peaks of Congo red at 500 nm and methyl orange at 425 nm sharply decrease with reaction time after the introduction of the as-prepared Au@CIAC-108-homo catalyst. Congo red and methyl orange are completely reduced to colorless leuco-forms within 75 seconds and 90 seconds (Fig. 4a and b), respectively. The conversion efficiency for Congo red and methyl orange reached 95.4% and 97.0%, respectively (Fig. S25 and S29†). In contrast, when Au/CIAC-108-homo and Au@CIAC-108-hetero were used as catalysts, much longer reaction time and lower conversion were observed (Fig. S26, S27, S30 and S31†). When the catalytic decompositions were conducted without any catalyst, the characteristic peaks of Congo red and methyl orange show negligible change after 90 min (Fig. S28 and S32†). Thus, it is reasonable to accept that the Au@CIAC-108-homo catalyst exhibits significantly enhanced catalytic activity for the decomposition reaction of organic dyes.

To further validate the general applicability of Au@CIAC-108-homo, the catalytic activity for the selective reduction of nitrobenzenes to the corresponding azobenzenes under visible-light irradiation was explored. The optimized reaction was performed in a methanol–dichloromethane (volume ratio = 1 : 2) mixture containing Au@CIAC-108-homo, nitrobenzene, and NaOH under visible-light irradiation. As shown in Table S2 and Fig. S33–S35,† the nitrobenzenes are selectively converted into the desired products with good-to-excellent yield. Particularly, 93.6% yield of the corresponding azobenzene product generated from the reduction of nitrobenzene was achieved (Table S2,† entry 1). The nitrobenzenes with –CH_3_ and –Cl at the *para*-position were converted to the corresponding azo products in 79.8% (Table S2,† entry 2) and 88.3% (Table S2,† entry 3) yields, respectively.

### Recyclability of Au@CIAC-108-homo

The durability and stability of Au@CIAC-108-homo are examined by successively adding fresh starting material (nitroarenes or organic dyes) into the reaction mixture after completion of the previous run, and no obvious loss in catalytic performance of Au@CIAC-108-homo is observed after 6 cycles (Fig. 3d, S32 and S33†). TEM measurement reveals that the size and morphology of Au@CIAC-108-homo have no noticeable changes even after the catalytic reaction (Fig. S34†). PXRD results demonstrated that the crystalline characteristics of CIAC-108 are preserved, and no obvious peaks of Au NPs are observed (Fig. S35†), indicating the remarkable stability of the Au@CIAC-108-homo catalyst. This demonstrates that the strong interactions between CIAC-108 and Au NPs could prevent the sintering and leaching of NPs. It is worth noting that the stability of Au@CIAC-108-homo is superior to that of some general supported Au NPs, such as Au/polyaniline,^70^ Au/dendrimer,^71^ and NAP-Mg-Au(0),^72^ for which the catalytic activity decreased with increasing cycles.

## Conclusions

In summary, coordination cages are demonstrated to be an excellent template to stabilize ultrafine Au NPs. The Au@CIAC-108 catalyst with Au NPs encapsulated inside the cavity of cage CIAC-108 exhibits smaller size (∼1.3 nm) and superior catalytic performances toward the 4-NPh reduction reaction and organic dye decomposition. The activities are much higher than those of the Au/CIAC-108 hybrid with larger Au NPs (∼4.7 nm) immobilized on the surface of the CIAC-108 host. Moreover, the Au@CIAC-108 catalyst shows excellent stability and durability. The intrinsic cavity of CIAC-108 possesses the ability to control the size of Au NPs, prevent them from aggregation, and maintain their excellent stability in solution. The present work may open a new frontier in the development of advanced catalysts through the encapsulation of ultrasmall MNPs in coordination cages.

## Experimental

### Materials and characterization

All the chemicals and solvents were purchased from commercial sources and used without further purification. FTIR spectra using KBr pellets were recorded on a BRUKER-EQUINOX-55 IR spectrophotometer. PXRD patterns were collected using a Rigaku Smartlab X-ray diffractometer with Cu Kα radiation in Bragg–Brentano mode. TEM images were recorded on a JEM-1400 Plus TEM microscope (80 kV). HRTEM and STEM images were recorded with a HAADF detector and the corresponding EDX elemental mapping was performed on an FEI Tecnai Osiris S/TEM (200 kV). XPS experiments were performed on an AVG Thermo ESCALAB 250 spectrometer (VG scientific) operated at 120 W. The UV-Vis absorption measurements were performed using a UV-2450 spectrophotometer (Shimadzu). Gas chromatography (GC) analysis was performed on an Agilent 7890B GC (the product yield was determined from the peak area in GC). ^1^H NMR spectra were recorded on a Variance VNMR 400 spectrometer in CDCl_3_. High resolution mass spectra (HRMS) were obtained on an AB 5800 MALDI-TOF/TOF and were recorded using electrospray ionization (ESI).

### Synthesis of CIAC-108

CIAC-108 was synthesized according to a modified literature method.^60^ CIAC-108 was obtained from a solvothermal reaction of a mixture of *p-tert*-butylthiacalix[4]arene (0.100 g, 0.14 mmol), CoCl_2_·6H_2_O (0.100 g, 0.40 mmol), isophthalonitrile (0.031 g, 0.24 mmol), sodium azide (0.036 g, 0.55 mmol), and CH_3_OH (13 mL) in a 20 mL Teflon-lined autoclave which was kept at 130 °C for 3 days and then slowly cooled to 20 °C at about 4 °C h^−1^. The crystals were isolated by filtration and then washed with methanol. Yield: *ca.* 45% based on calixarene.

### Synthesis of catalysts

#### Synthesis of the Au@CIAC-108 catalyst

In a typical synthesis, 80 mg of activated CIAC-108 powder was suspended in 20 mL of ethanol with vigorous stirring under ambient conditions. After constant vigorous stirring overnight, the ethanol solution of HAuCl_4_·4H_2_O (240 μL, 2 mg mL^−1^) was added and the resulting mixture was continuously stirred for 3 h. Finally, 0.25 mL of freshly prepared aqueous NaBH_4_ (2.7 M) solution was added quickly and then kept under stirring for 3 h. The synthesized sample was gathered by centrifugation and washed with ethanol. The obtained solid Au@CIAC-108 sample was dried in air at 60 °C for 2 h and used for the catalytic reactions.

#### Synthesis of the Au/CIAC-108 catalyst

The synthetic procedure was similar to that of Au@CIAC-108 except that the corresponding activated CIAC-108 powder (78 mg), the ethanol solution of HAuCl_4_·4H_2_O (350 μL, 2 mg mL^−1^), and freshly prepared aqueous NaBH_4_ (0.8 mL, 2.7 M) solution were used.

#### Synthesis of Au/(H_4_TC4A–IPN–NaN_3_ mixture)

The synthetic procedure was similar to that of Au@CIAC-108 except that *p-tert*-butylthiacalix[4]arene (H_4_TC4A, 67 mg), isophthalonitrile (IPN, 32 mg) and sodium azide (NaN_3_, 28 mg) were used in place of CIAC-108.

### Catalytic activity characterization

#### Catalytic hydrogenation of 4-NPh by the Au@CIAC-108-homo catalyst

Generally, the reaction was performed under ambient conditions. First, a mixture of 40 μL of 4-NPh (14.65 mmol L^−1^) and 2 mL of Au@CIAC-108-homo catalyst (0.00000251 mmol Au) in a CH_3_OH and CH_2_Cl_2_ mixture (volume ratio = 1 : 2) was mixed in a quartz cell. Then, 10 mg of NaBH_4_ (0.264 mmol) was introduced to the solution. The initial molar ratio of catalyst/4-NPh/NaBH_4_ was adjusted to 1/234/105 214. After introducing the NaBH_4_, the color of the 4-NPh solution gradually faded from bright yellow to colorless as the reaction continued. The conversion of 4-NPh to 4-aminophenol was monitored by recording the UV-Vis spectra at short intervals in the range 250–500 nm. The rate constants of the reduction process were determined through measuring the change in absorbance at *λ* = 400 nm as a function of time.

#### Catalytic hydrogenation of 4-NPh by the Au/CIAC-108-homo catalyst

The catalytic procedure was similar to that of Au@CIAC-108-homo except that Au@CIAC-108-homo (0.00000251 mmol Au) was replaced by Au/CIAC-108-homo (0.00000375 mmol Au). The initial molar ratio of catalyst/4-NPh/NaBH_4_ was adjusted to 1/156/70 343.

#### Catalytic hydrogenation of 4-NPh by the Au@CIAC-108-hetero catalyst

The catalytic procedure was similar to that of Au@CIAC-108-homo (0.00000251 mmol Au) except that the CH_3_OH and CH_2_Cl_2_ solvent mixture was replaced by sole CH_3_OH solvent.

#### Catalytic decomposition of Congo red by the Au@CIAC-108-homo catalyst

Typically, a mixture of 80 μL of Congo red (6.67 mmol L^−1^) and 2 mL of Au@CIAC-108-homo catalyst (0.00000251 mmol Au) in a CH_3_OH and CH_2_Cl_2_ mixture (volume ratio = 1 : 2) was mixed in a quartz cell. 6 mg of NaBH_4_ (0.159 mmol) was subsequently introduced to the solution. The color of Congo red solution gradually faded from bright red to colorless as the reaction proceeded. The catalytic reactions were detected using UV-Vis spectra. Based on the changes in the intensity at *λ* = 500 nm as a function of time, the rate constant of the catalytic decomposition was monitored using UV-Vis spectra.

#### Catalytic decomposition of Congo red by the Au/CIAC-108-homo catalyst

The catalytic procedure was similar to that of Au@CIAC-108-homo except that Au@CIAC-108-homo (0.00000251 mmol Au) was replaced by Au/CIAC-108-homo (0.00000375 mmol Au).

#### Catalytic decomposition of methyl orange by Au@CIAC-108-homo and Au/CIAC-108-homo catalysts

The reduction procedure was similar to that of methyl orange. The color of methyl orange solution gradually faded from orange to colorless as the reaction continued. On the basis of the changes in the intensity at *λ* = 425 nm as a function of time, the rate constant of the reduction process was determined.

#### Catalytic decomposition of methyl orange and Congo red by the Au@CIAC-108-hetero catalyst

The reduction procedure was similar to that of Au@CIAC-108-homo except for the replacement of the CH_3_OH and CH_2_Cl_2_ solvent mixture by sole methanol solvent.

## Author contributions

X. H., S. W. and Q. X. conceived and designed the project. X. H. and S. W. performed the experiments and characterization. X. H. and H. P. analysed the experimental results. X. H. and Q. X. wrote the manuscript with input from the other authors.

## Conflicts of interest

There are no conflicts to declare.

## Supplementary Material

SC-013-D1SC05407D-s001
