# Supplementary material for: A coordination cage hosting ultrafine and highly catalytically active gold nanoparticles
Source: Chem Sci. 2021 Dec 7;13(2):461–8. doi: 10.1039/d1sc05407d (PMC8729796; doi:10.1039/d1sc05407d)
Supplement: SC-013-D1SC05407D-s001 [file SC-013-D1SC05407D-s001.pdf]

Electronic Supplementary Material (ESI) for Chemical Science.
This journal is © The Royal Society of Chemistry 2021

Coordination cage hosting ultrafine and highly catalytically active gold nanoparticles

Xinxin Hang,[a] Shentang Wang,*[b] Huan Pang,*[a] and Qiang Xu*[a,c,d]

[a]School of Chemistry and Chemical Engineering, Institute for Innovative Materials and Energy, Yangzhou University, Yangzhou, Jiangsu 225002, China

[b]School of Chemistry and Chemical Engineering, and Chongqing Key Laboratory of Soft-Matter Material Chemistry and Function Manufacturing, Southwest University, Chongqing 400715, China

[c]Department of Materials Science and Engineering and SUSTech Academy for Advanced Interdisciplinary Studies, Southern University of Science and Technology (SUSTech), Shenzhen 518055, China.

[d]Institute for Integrated Cell-Material Sciences (iCeMS), Kyoto University, Yoshida, Sakyo-ku, Kyoto 606-8501, Japan

Table of Contents

Experimental Details S1

Supporting Figures and Table..S4

References..S26

Experimental Details

Catalytic hydrogenation of 2-NPh by Au@CIAC-108-*homo* catalyst. A mixture of 45 µL of 2-NPh (21.57 mmol L^{-1}) and 2 mL of Au@CIAC-108-*homo* catalyst (0.00000251 mmol Au) in CH_3OH and CH_2Cl_2 mixture (volume ratio = 1:2) was mixed in a quartz cell. 10 mg of $NaBH_4$ (0.264 mmol) was subsequently introduced to the solution. The initial molar ratio of catalyst/2-NPh/$NaBH_4$ was adjusted to 1/387/105214. After introducing the $NaBH_4$, the color of the 2-NPh solution gradually faded from bright yellow to colorless as the reaction continued. The conversion of 2-NPh to 2-aminophenol was monitored by recording the UV-Vis spectra at short intervals in the range 250-500 nm. On the basis of the change in the intensity at λ = 420 nm as a function of time, the rate constants of the catalytic hydrogenation of 2-NPh were determined.

Catalytic hydrogenation of 2-NPh by Au/CIAC-108-*homo* catalyst. The catalytic procedure was similar to that of Au@CIAC-108-*homo* except Au@CIAC-108-*homo* (0.00000251 mmol Au) was replaced by Au/CIAC-108-*homo* (0.00000375 mmol Au). The initial molar ratio of catalyst/2-NPh/$NaBH_4$ was adjusted to 1/259/70343.

Catalytic hydrogenation of 2-NAn by Au@CIAC-108-*homo* catalyst. Generally, the reaction was carried out under ambient conditions. First, 50 µL of 2-NAn (21.72 mmol L^{-1}) and 2 mL of Au@CIAC-108-*homo* catalyst (0.00000251 mmol Au) in CH_3OH and CH_2Cl_2 mixture (volume ratio = 1:2) was mixed in a quartz cell. 10 mg of $NaBH_4$ (0.264 mmol) was subsequently introduced to the solution. The initial molar ratio of catalyst/2-NAn/$NaBH_4$ was adjusted to 1/433/105214. After introducing the catalyst, the color of the 2-NAn solution gradually faded from bright yellow to colorless as the reaction continued. The conversion of 2-NAn to 2-phenylenediamine was monitored by recording the UV-Vis spectra at short intervals in the range 250-500 nm. The rate constants of the reduction process were determined through measuring the change in absorbance at λ = 400 nm as a function of time.

Catalytic hydrogenation of 2-NAn by Au/CIAC-108-*homo* catalyst. The catalytic procedure was similar to that of Au@CIAC-108-*homo* except Au@CIAC-108-*homo* (0.00000251 mmol Au) was replaced by Au/CIAC-108-*homo* (0.00000375 mmol Au).

The initial molar ratio of catalyst/2-NAn/$NaBH_4$ was adjusted to 1/289/70343.

Catalytic hydrogenation of 3-NAn by Au@CIAC-108-*homo* catalyst. A mixture of 45 μL of 2-NPh (21.57 mmol L^{-1}) and 2 mL of Au@CIAC-108-*homo* catalyst (0.00000251 mmol Au) in CH_3OH and CH_2Cl_2 mixture (volume ratio = 1:2) was mixed in a quartz cell. 10 mg of $NaBH_4$ (0.264 mmol) was subsequently introduced to the solution. The initial molar ratio of catalyst/3-NAn/$NaBH_4$ was adjusted to 1/387/105214. The color of the 3-NAn solution gradually faded from bright yellow to colorless as the reaction continued. The conversion of 3-NAn to 3-phenylenediamine was monitored by recording the UV-Vis spectra at short intervals in the range 250-500 nm. On the basis of the change in the intensity at λ = 370 nm as a function of time, the rate constants of the catalytic hydrogenation of 3-NAn were determined.

Catalytic hydrogenation of 3-NAn by Au/CIAC-108-*homo* catalyst. The catalytic procedure was similar to that of Au@CIAC-108-*homo* except Au@CIAC-108-*homo* (0.00000251 mmol Au) was replaced by Au/CIAC-108-*homo* (0.00000375 mmol Au). The initial molar ratio of catalyst/3-NAn/$NaBH_4$ was adjusted to 1/259/70343.

Catalytic hydrogenation of 4-NAn by Au@CIAC-108-*homo* catalyst. Generally, the reaction was carried out under ambient conditions. First, 80 μL of 4-NAn (21.72 mmol L^{-1}) and 2 mL of Au@CIAC-108-*homo* catalyst (0.00000251 mmol Au) in CH_3OH and CH_2Cl_2 mixture (volume ratio = 1:2) was mixed in a quartz cell. 10 mg of $NaBH_4$ (0.264 mmol) was subsequently introduced to the solution. The initial molar ratio of catalyst/4-NAn/$NaBH_4$ was adjusted to 1/693/105214. After introducing the $NaBH_4$, the color of the 4-NAn solution gradually faded from bright yellow to colorless as the reaction continued. The conversion of 4-NAn to 4-phenylenediamine was monitored by recording the UV-Vis spectra at short intervals in the range 250-500 nm. The rate constants of the reduction process were determined through measuring the change in absorbance at λ = 370 nm as a function of time.

To be noted, the catalytic reaction of 2-NAn and 4-NAn by Au@CIAC-108-*homo* with 5 mg of $NaBH_4$ (0.132 mmol) was also performed. The initial molar ratio of catalyst/4-NAn/$NaBH_4$ was adjusted to 1/387/52607.

Catalytic hydrogenation of 4-NAn by Au/CIAC-108-*homo* catalyst. The catalytic

procedure was similar to that of Au@CIAC-108-*homo* except Au@CIAC-108-*homo* (0.00000251 mmol Au) was replaced by Au/CIAC-108-*homo* (0.00000375 mmol Au). The initial molar ratio of catalyst/3-NAn/$NaBH_4$ was adjusted to 1/463/70343.

Catalytic hydrogenation of 2-NPh, 2-NAn, 3-NAn, and 4-NAn by Au@CIAC-108-*heter* catalyst. The catalytic procedure was similar to that of Au@CIAC-108-*homo* (0.00000251 mmol Au) except the CH_3OH and CH_2Cl_2 solvent mixture was replaced by sole CH_3OH solvent.

Catalytic reduction of nitrobenzenes to azobenzenes by Au@CIAC-108-*homo* catalyst. A mixture of Au@CIAC-108-*homo* (0.00000251 mmol Au), nitrobenzene (120 mL, 1.18 mmol), NaOH (120 mg, 3 mmol), CH_3OH (2 mL) and CH_2Cl_2 (4 mL) was stirred at room temperature under visible light irradiation (λ = 435 nm, 300 W Xenon with a power density of 2.5 W/cm^2) for 12 h in air (monitored by GC) to obtain the corresponding product.

Durability test of Au@CIAC-108-*homo* catalyst

The durability of Au@CIAC-108-*homo* is examined by successively adding fresh starting material (nitroarenes or organic dyes) into the reaction mixture after completion of the previous run. Such test was performed for 6 cycles at room temperature.

Stability test of Au@CIAC-108-*homo* catalyst

After the catalytic reaction, a solution of Au@CIAC-108-*homo* catalyst with dilution was drop-cast onto carbon-coated copper grids and dried for the TEM measurements. The Au@CIAC-108-*homo* catalyst after 6 cycles of catalytic reaction was washed by CH_3OH and CH_2Cl_2 solvent and dried under vacuum for PXRD analysis.

Supporting Figures and Tables

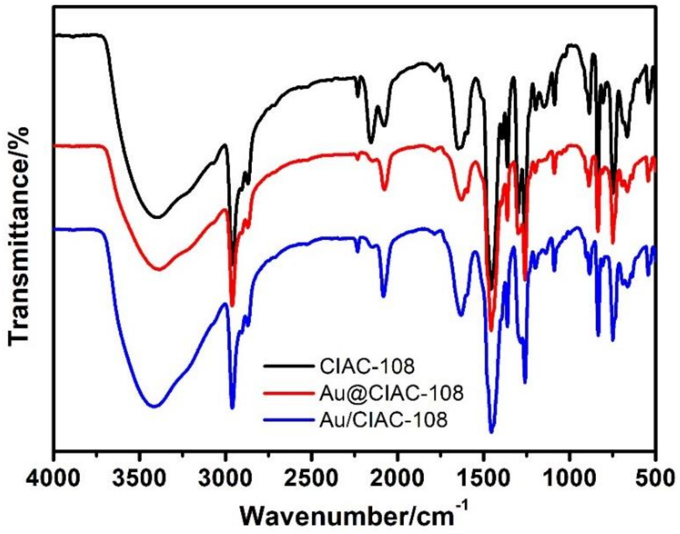

Fig. S1 FTIR spectra of CIAC-108, Au@CIAC-108 and Au/CIAC-108.

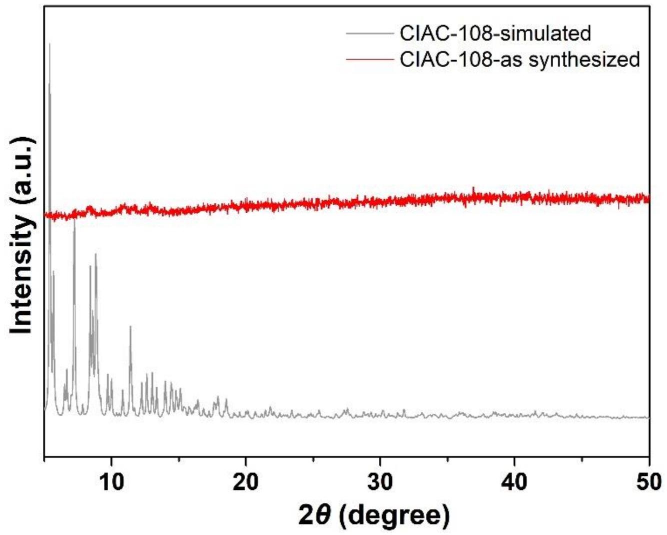

Fig. S2 PXRD patterns of simulated CIAC-108 (the crystal X-ray diffraction data collected in the range 5-50º) and as-synthesized CIAC-108.

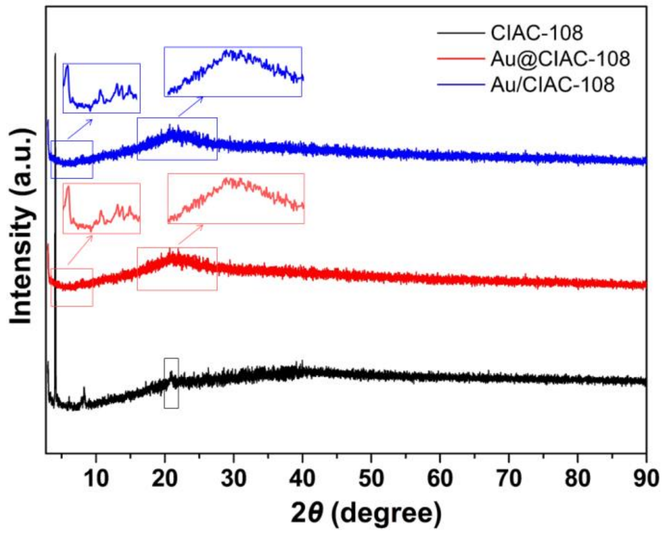

Fig. S3 PXRD patterns of as-synthesized CIAC-108, Au@CIAC-108, and Au/CIAC-108 (the data collected in the range 3-90º).

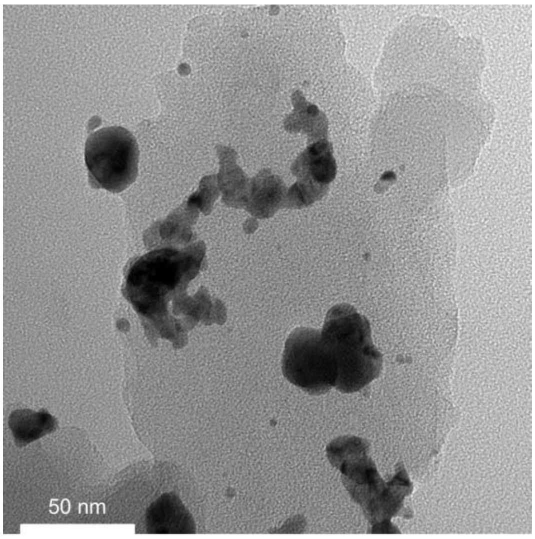

Fig. S4 TEM image of the Au/(H_4TC4A-IPN-NaN_3 mixture).

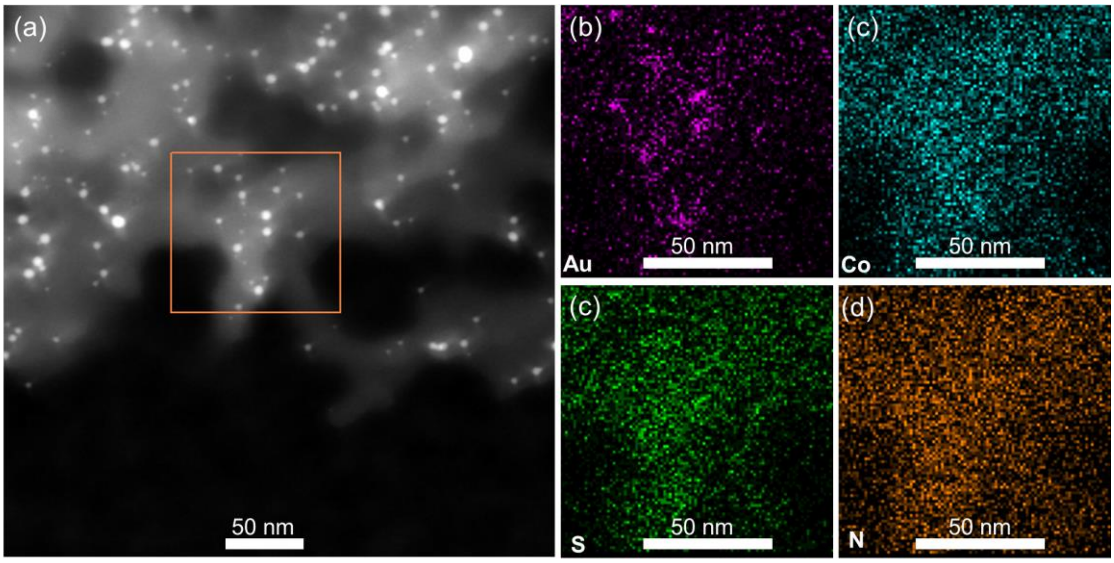

Fig. S5 (a) HAADF-STEM image of Au/CIAC-108 and (b-e) EDS mapping images corresponding to the image in (a).

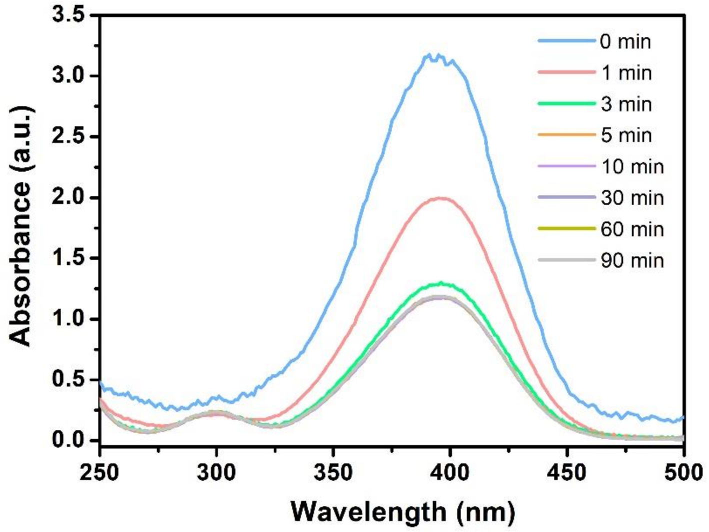

Fig. S6 UV-vis spectra of reduction of 4-NPh by the Au/CIAC-108-*homo* catalyst in CH_3OH/ CH_2Cl_2 (v/v=1/2) in the presence of 10 mg $NaBH_4$.

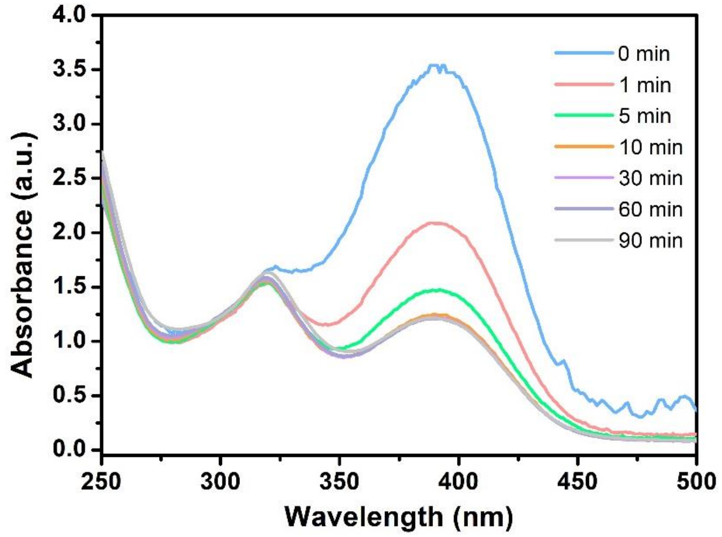

Fig. S7 UV-vis spectra of hydrogenation of 4-NPh by the Au@CIAC-108-*heter* catalyst in CH_3OH in the presence of 10 mg $NaBH_4$.

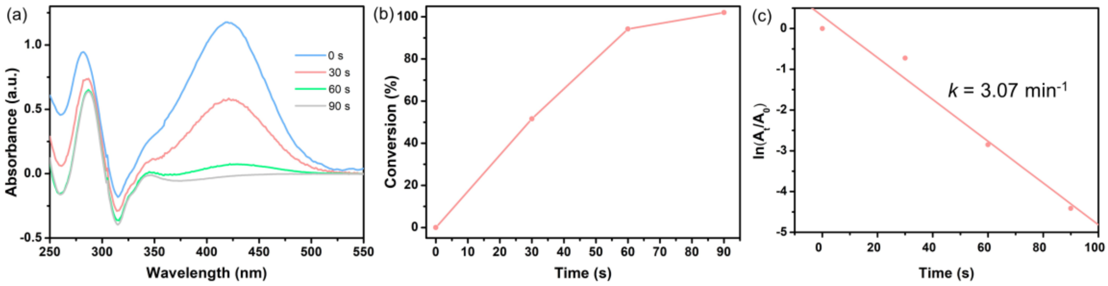

Fig. S8 (a) UV-vis spectra of reduction of 2-NPh by the Au@CIAC-108-*homo* catalyst in CH_3OH/CH_2Cl_2 (v/v=1/2) in the presence of 10 mg $NaBH_4$. (b) Catalytic conversion of 2-NPh over Au@CIAC-108-*homo* at 298 K. (c) Plot of $\ln(A_t/A_0)$ of absorbance of 2-NPh at 420 nm obtained from the spectra in (a) versus time for the hydrogenation of 2-NPh catalyzed by the Au@CIAC-108-*homo*.

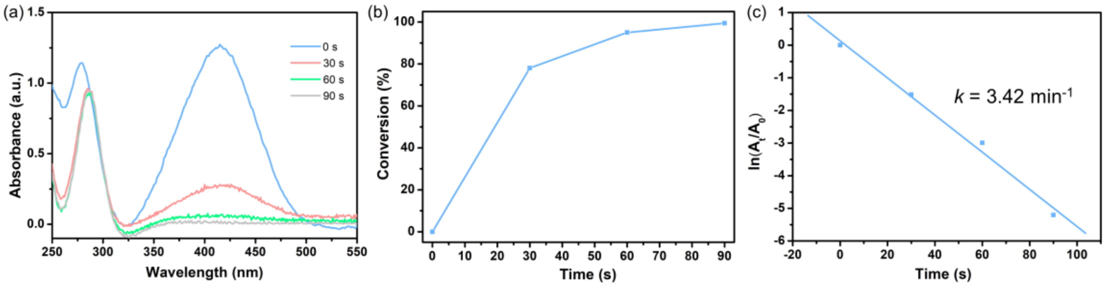

Fig. S9 (a) UV-vis spectra of reduction of 2-NPh by the Au/CIAC-108-*homo* catalyst in CH_3OH/CH_2Cl_2 (v/v=1/2) in the presence of 10 mg $NaBH_4$. (b) Catalytic conversion of 2-NPh over Au/CIAC-108-*homo* at 298 K. (c) Plot of $\ln(A_t/A_0)$ of absorbance of 2-NPh at 420 nm obtained from the spectra in (a) versus time for the hydrogenation of 2-NPh catalyzed by the Au/CIAC-108-*homo*.

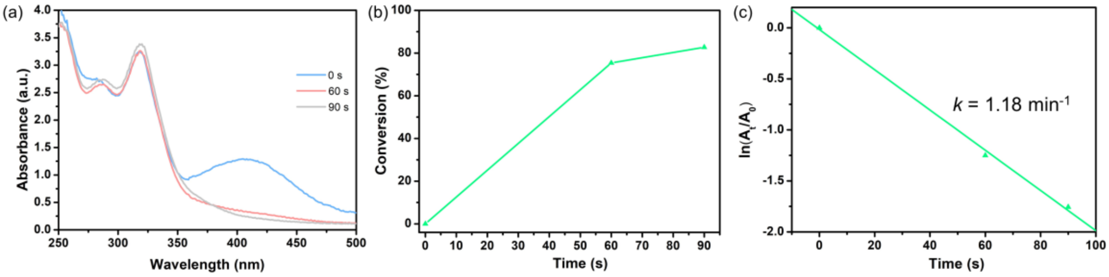

Fig. S10 (a) UV-vis spectra of reduction of 2-NPh by the Au@CIAC-108-*heter* catalyst in CH_3OH in the presence of 10 mg $NaBH_4$. (b) Catalytic conversion of 2-NPh over Au@CIAC-108-*heter* at 298 K. (c) Plot of $\ln(A_t/A_0)$ of absorbance of 2-NPh at 420 nm obtained from the spectra in (a) versus time for the hydrogenation of 2-NPh catalyzed by the Au@CIAC-108-*heter*.

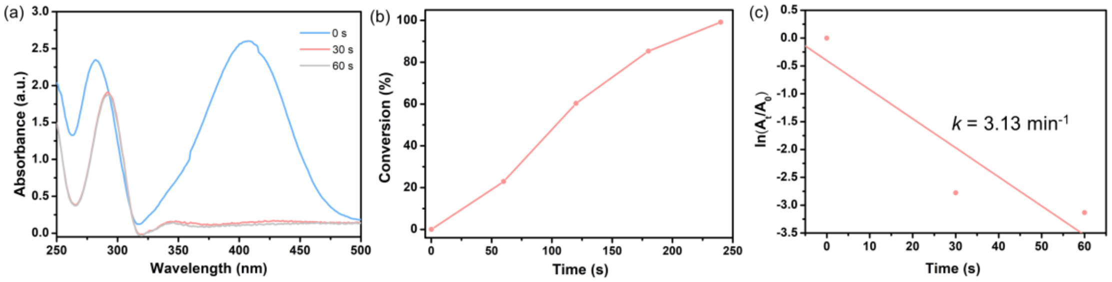

Fig. S11 (a) UV-vis spectra of reduction of 2-NAn by the Au@CIAC-108-*homo* catalyst in CH_3OH/CH_2Cl_2 (v/v=1/2) in the presence of 10 mg $NaBH_4$. (b) Catalytic conversion of 2-NAn over Au@CIAC-108-*homo* at 298 K. (c) Plot of ln(A_t/A_0) of absorbance of 2-NAn at 400 nm obtained from the spectra in (a) versus time for the hydrogenation of 2-NAn catalyzed by the Au@CIAC-108-*homo*.

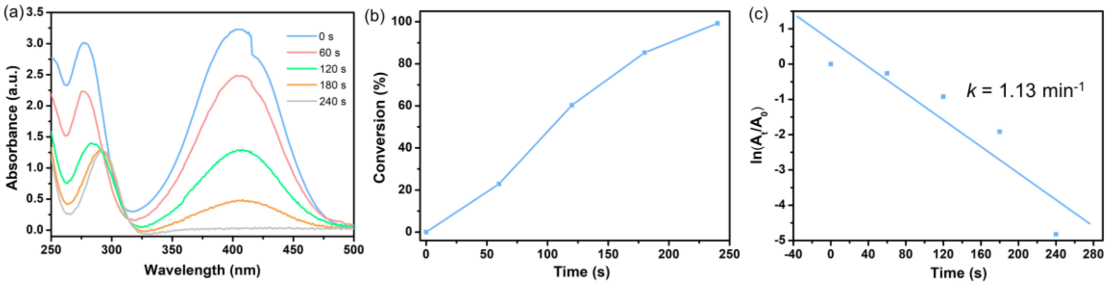

Fig. S12 (a) UV-vis spectra of reduction of 2-NAn by the Au/CIAC-108-*homo* catalyst in CH_3OH/CH_2Cl_2 (v/v=1/2) in the presence of 10 mg $NaBH_4$. (b) Catalytic conversion of 2-NAn over Au/CIAC-108-*homo* at 298 K. (c) Plot of ln(A_t/A_0) of absorbance of 2-NAn at 400 nm obtained from the spectra in (a) versus time for the hydrogenation of 2-NAn catalyzed by the Au/CIAC-108-*homo*.

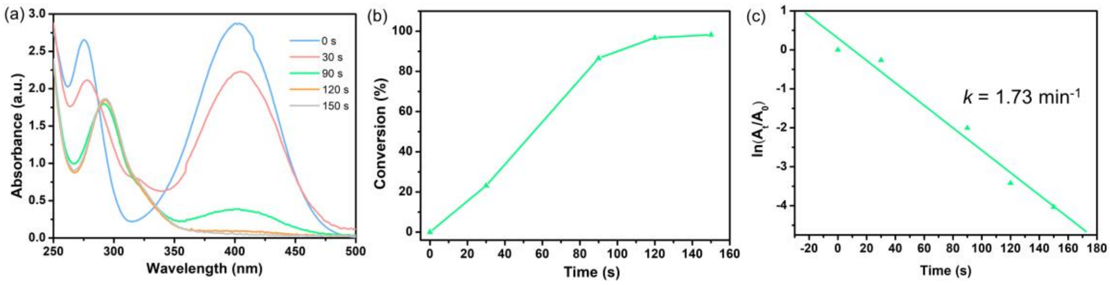

Fig. S13 (a) UV-vis spectra of reduction of 2-NAn by the Au@CIAC-108-*heter* catalyst in CH_3OH in the presence of 10 mg $NaBH_4$. (b) Catalytic conversion of 2-NAn over Au@CIAC-108-*heter* at 298 K. (c) Plot of ln(A_t/A_0) of absorbance of 2-NAn at 400 nm obtained from the spectra in (a) versus time for the hydrogenation of 2-NAn catalyzed by the Au@CIAC-108-*heter*.

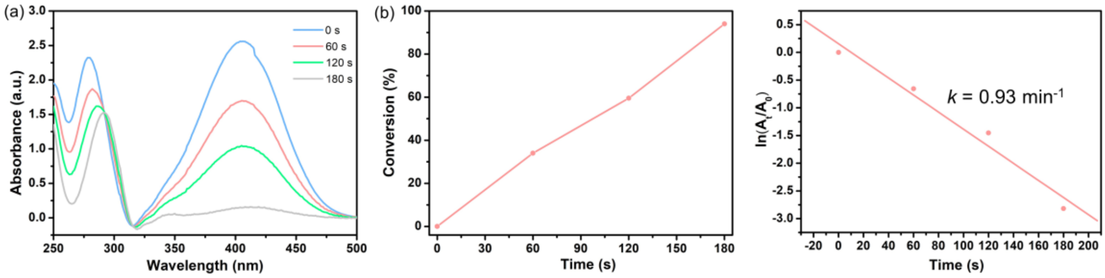

Fig. S14 (a) UV-vis spectra of reduction of 2-NAn by the Au@CIAC-108-*homo* catalysts in the presence of 5 mg $NaBH_4$ at 298 K. (b) Plots of $\ln(A_t/A_0)$ of absorbance of 2-NAn at 400 nm obtained from the spectra in (a) versus time for the hydrogenation of 2-NAn catalyzed by the Au@CIAC-108-*homo* catalysts.

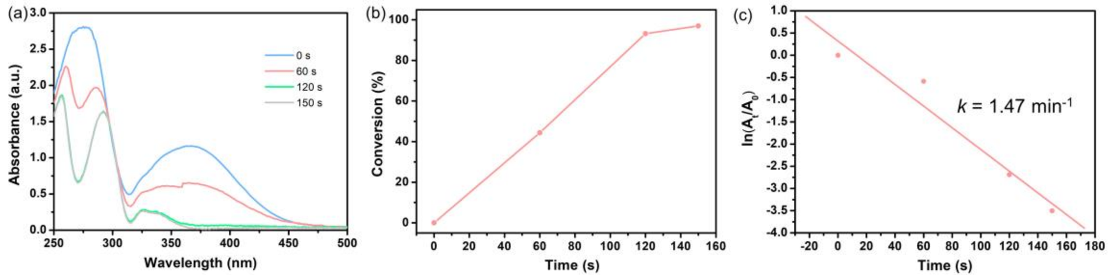

Fig. S15 (a) UV-vis spectra of reduction of 3-NAn by the Au@CIAC-108-*homo* catalyst in CH_3OH/CH_2Cl_2 (v/v=1/2) in the presence of 10 mg $NaBH_4$. (b) Catalytic conversion of 3-NAn over Au@CIAC-108-*homo* at 298 K. (c) Plot of $\ln(A_t/A_0)$ of absorbance of 3-NAn at 370 nm obtained from the spectra in (a) versus time for the hydrogenation of 3-NAn catalyzed by the Au@CIAC-108-*homo*.

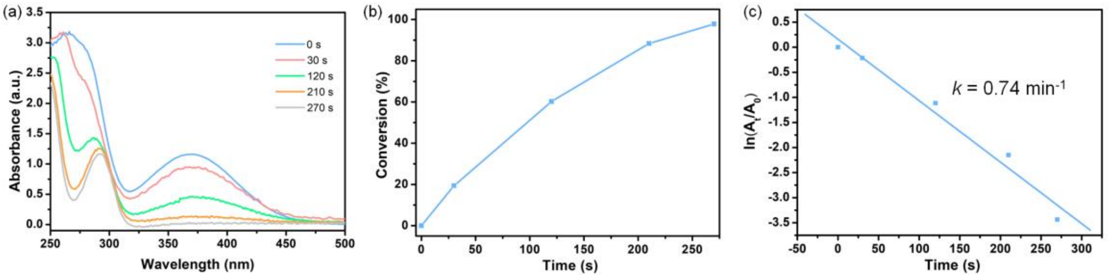

Fig. S16 (a) UV-vis spectra of reduction of 3-NAn by the Au/CIAC-108-*homo* catalyst in CH_3OH/CH_2Cl_2 (v/v=1/2) in the presence of 10 mg $NaBH_4$. (b) Catalytic conversion of 3-NAn over Au/CIAC-108-*homo* at 298 K. (c) Plot of $\ln(A_t/A_0)$ of absorbance of 3-NAn at 370 nm obtained from the spectra in (a) versus time for the hydrogenation of 3-NAn catalyzed by the Au/CIAC-108-*homo*.

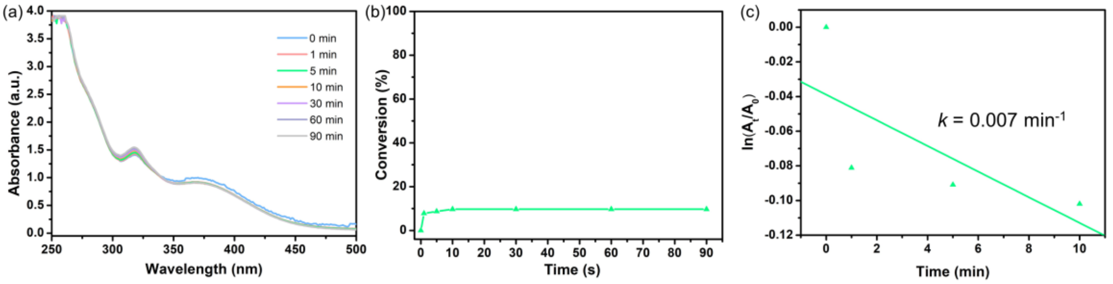

Fig. S17 (a) UV-vis spectra of reduction of 3-NAn by the Au@CIAC-108-*heter* catalyst in CH_3OH in the presence of 10 mg $NaBH_4$. (b) Catalytic conversion of 3-NAn over Au@CIAC-108-*heter* at 298 K. (c) Plot of $\ln(A_t/A_0)$ of absorbance of 3-NAn at 370 nm obtained from the spectra in (a) versus time for the hydrogenation of 3-NAn catalyzed by the Au@CIAC-108-*heter* (in the initial 10 mins).

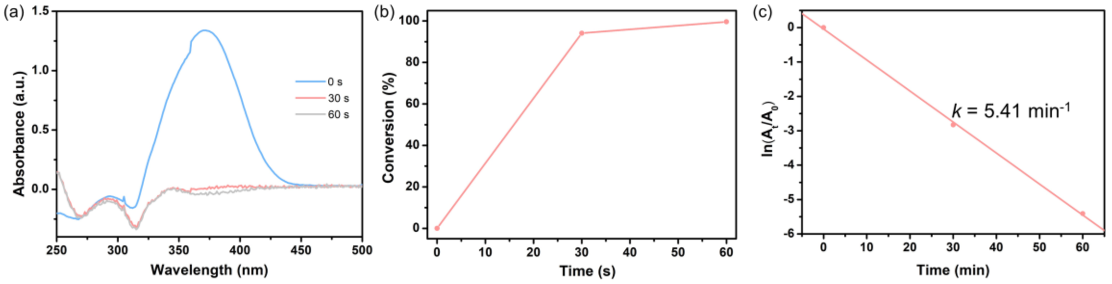

Fig. S18 (a) UV-vis spectra of reduction of 4-NAn by the Au@CIAC-108-*homo* catalyst in CH_3OH/CH_2Cl_2 (v/v=1/2) in the presence of 10 mg $NaBH_4$. (b) Catalytic conversion of 4-NAn over Au@CIAC-108-*homo* at 298 K. (c) Plot of ln(A_t/A_0) of absorbance of 4-NAn at 370 nm obtained from the spectra in (a) versus time for the hydrogenation of 4-NAn catalyzed by the Au@CIAC-108-*homo*.

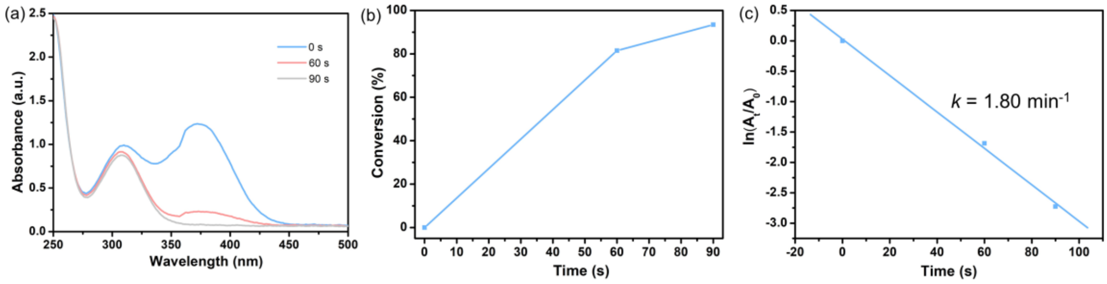

Fig. S19 (a) UV-vis spectra of reduction of 4-NAn by the Au/CIAC-108-*homo* catalyst in CH_3OH/CH_2Cl_2 (v/v=1/2) in the presence of 10 mg $NaBH_4$. (b) Catalytic conversion of 4-NAn over Au/CIAC-108-*homo* at 298 K. (c) Plot of ln(A_t/A_0) of absorbance of 4-NAn at 370 nm obtained from the spectra in (a) versus time for the hydrogenation of 4-NAn catalyzed by the Au/CIAC-108-*homo*.

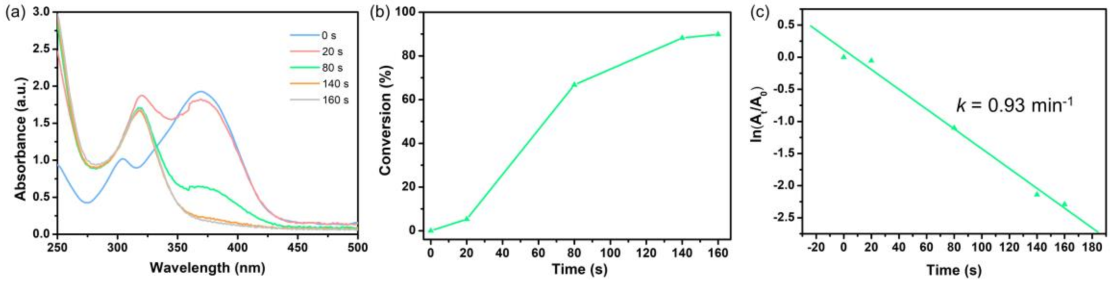

Fig. S20 (a) UV-vis spectra of reduction of 4-NAn by the Au@CIAC-108-*heter* catalyst in CH_3OH in the presence of 10 mg $NaBH_4$. (b) Catalytic conversion of 4-NAn over Au@CIAC-108-*heter* at 298 K. (c) Plot of ln(A_t/A_0) of absorbance of 4-NAn at 370 nm obtained from the spectra in (a) versus time for the hydrogenation of 4-NAn catalyzed by the Au@CIAC-108-*heter*.

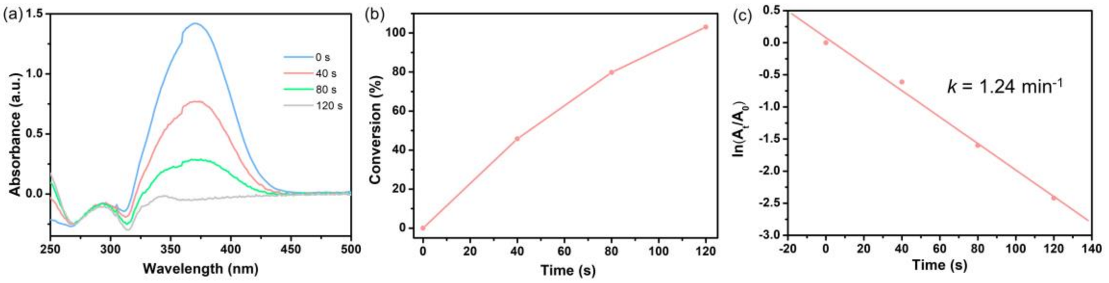

Fig. S21 (a) UV-vis spectra of reduction of 4-NAn by the Au@CIAC-108-*homo* catalyst in CH_3OH/CH_2Cl_2 (v/v=1/2) in the presence of 5 mg $NaBH_4$. (b) Catalytic conversion of 4-NAn over Au@CIAC-108-*homo* at 298 K. (c) Plot of $\ln(A_t/A_0)$ of absorbance of 4-NAn at 370 nm obtained from the spectra in (a) versus time for the hydrogenation of 4-NAn catalyzed by the Au@CIAC-108-*homo*.

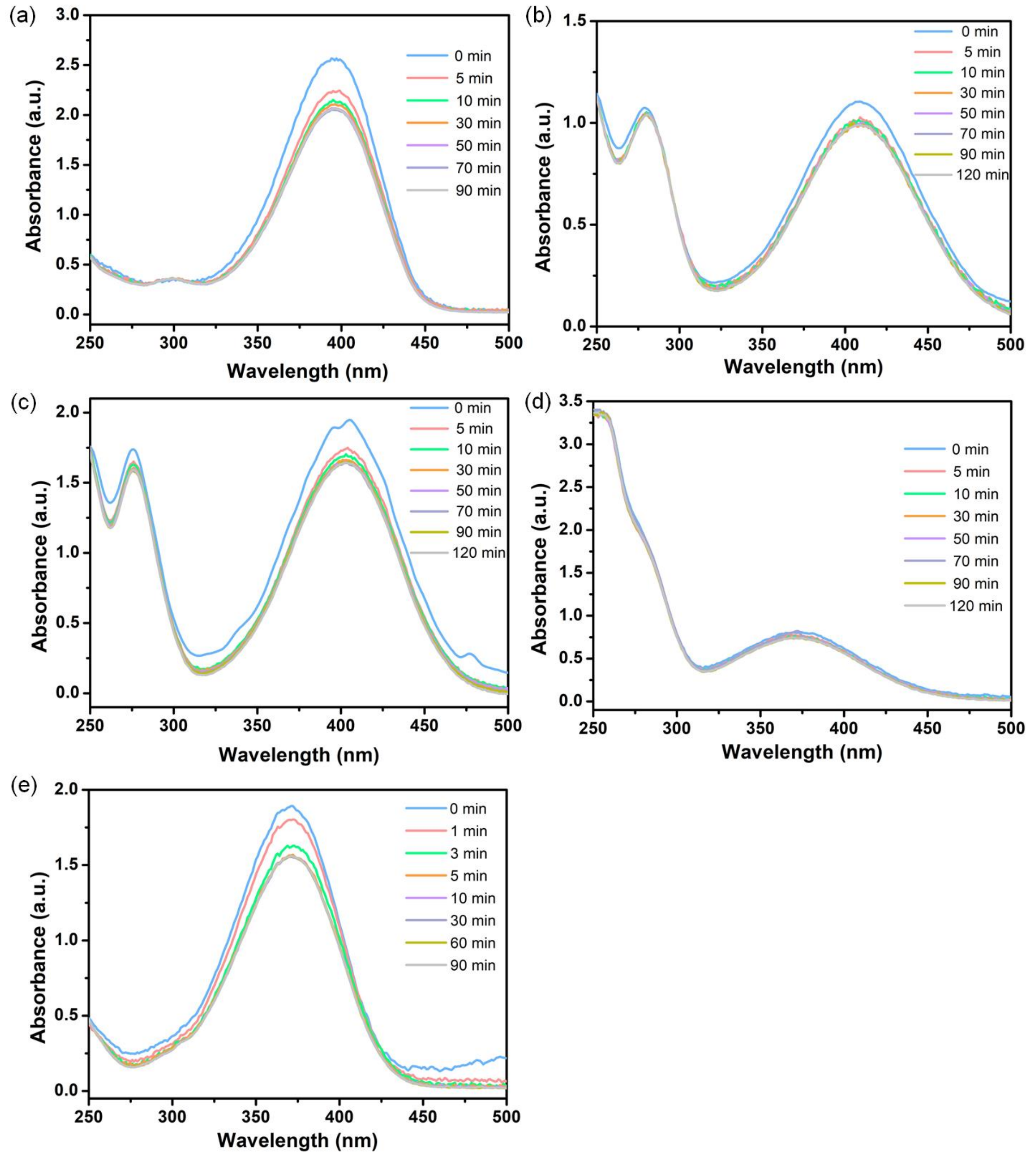

Fig. S22 UV-vis absorption spectra of (a)4-NPh, (d)2-NPh, (c)2-NAn, (d)3-NAn, and (e)4-NAn in CH_3OH/CH_2Cl_2 (v/v=1/2) in the presence of 10 mg $NaBH_4$ without catalyst. The absorption spectra show no significant changes in 90 min (a,e) or 120 min (b-d).

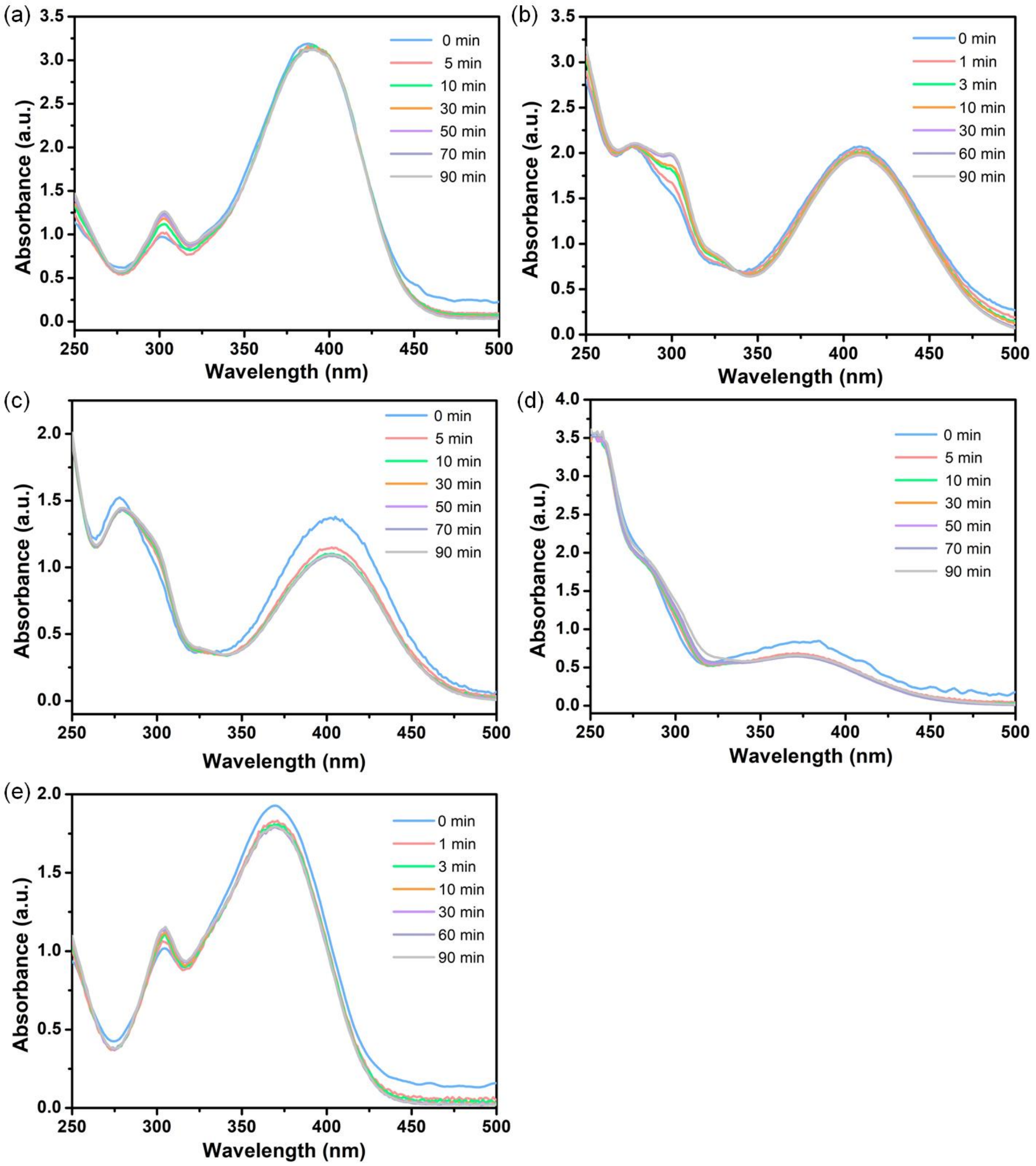

Fig. S23 UV-vis absorption spectra of (a)4-NPh, (d)2-NPh, (c)2-NAn, (d)3-NAn, and (e)4-NAn in CH_3OH/CH_2Cl_2 (v/v=1/2) in the presence of 10 mg $NaBH_4$ with the Au/(H_4TC4A-IPN-NaN_3 mixture) catalyst. The absorption spectra show no significant changes in 90 min.

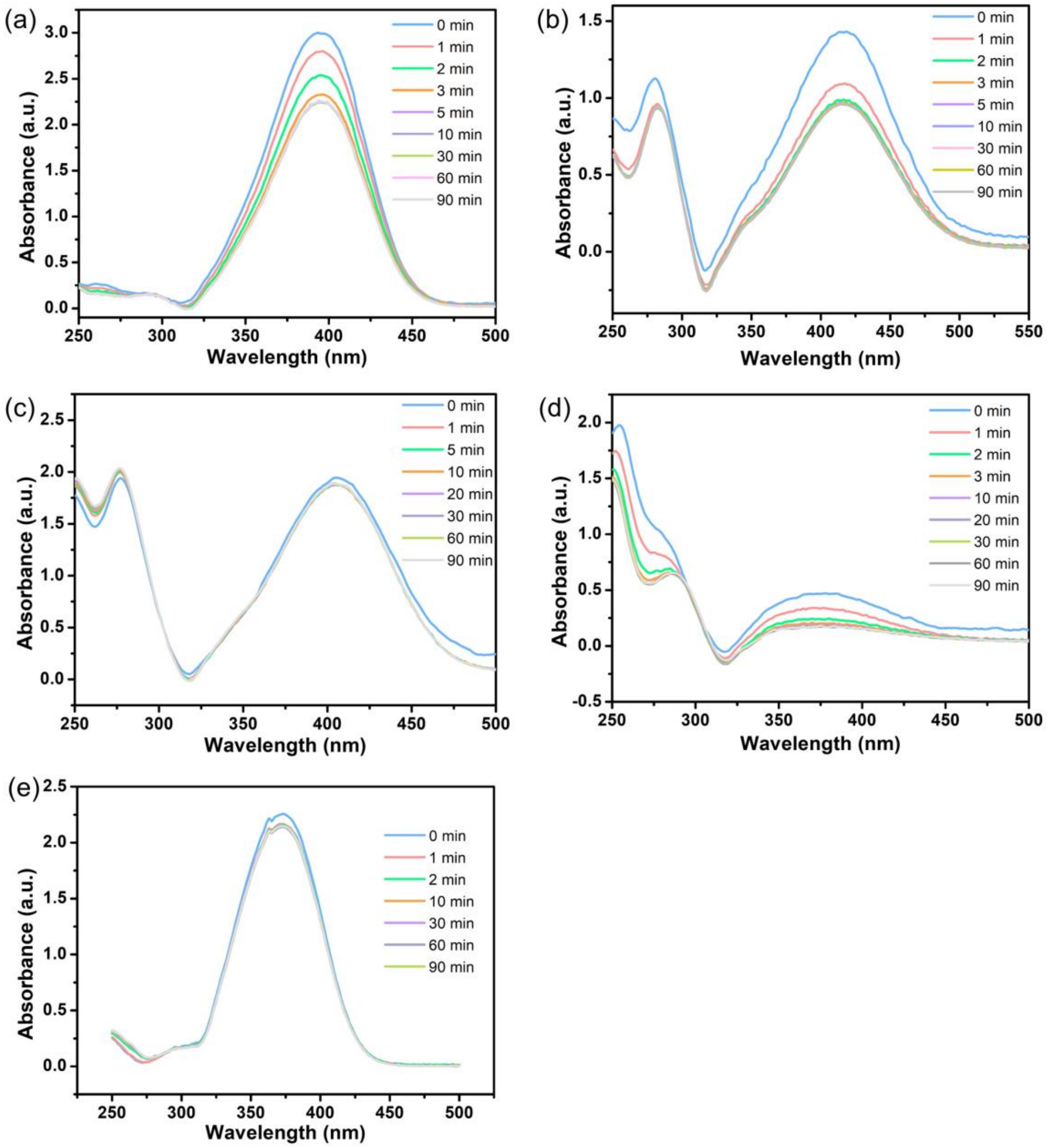

Fig. S24 UV-vis absorption spectra of (a)4-NPh, (d)2-NPh, (c)2-NAn, (d)3-NAn, and (e)4-NAn in CH_3OH/CH_2Cl_2 (v/v=1/2) in the presence of 10 mg $NaBH_4$ with the CIAC-108 catalyst. The absorption spectra show no significant changes in 90 min.

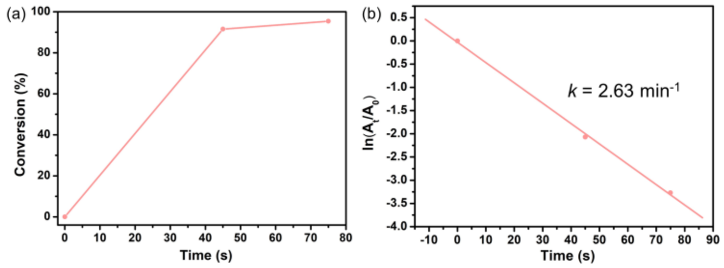

Fig. S25 (a) Catalytic conversion of Congo red over Au@CIAC-108-*homo* in CH_3OH/CH_2Cl_2 (v/v=1/2) in the presence of 10 mg $NaBH_4$ at 298 K. (b) Plot of $\ln(A_t/A_0)$ of absorbance of Congo red at 495 nm obtained from the spectra versus time for the hydrogenation of Congo red catalyzed by the Au@CIAC-108-*homo* catalyst.

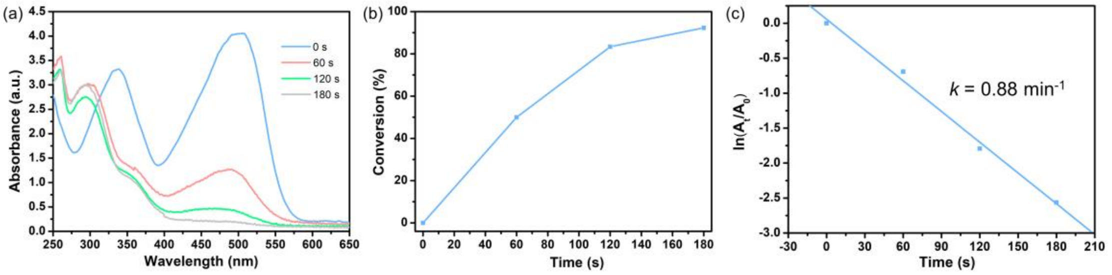

Fig. S26 (a) UV-vis spectra of reduction of Congo red by the Au/CIAC-108-*homo* catalyst in CH_3OH/CH_2Cl_2 (v/v=1/2) in the presence of 10 mg $NaBH_4$. (b) Catalytic conversion of Congo red over Au/CIAC-108-*homo* at 298 K. (c) Plot of $\ln(A_t/A_0)$ of absorbance of Congo red at 495 nm obtained from the spectra in (a) versus time for the hydrogenation of Congo red catalyzed by the Au/CIAC-108-*homo* catalyst.

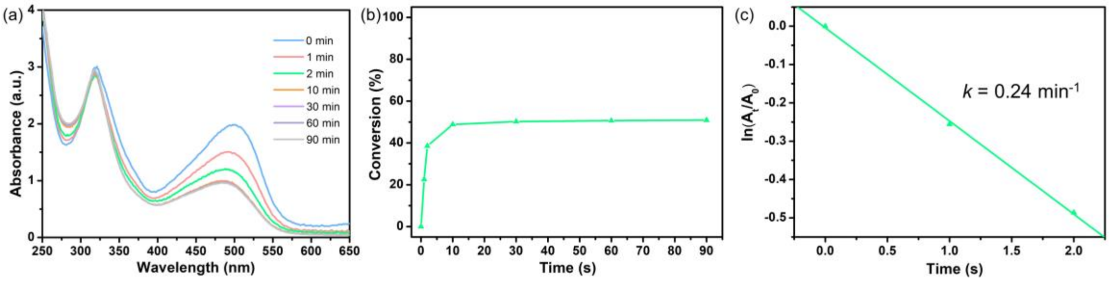

Fig. S27 (a) UV-vis spectra of reduction of Congo red by the Au@CIAC-108-*heter* catalyst in CH_3OH in the presence of 10 mg $NaBH_4$. (b) Catalytic conversion of Congo red over Au@CIAC-108-*heter* at 298 K. (c) Plot of $\ln(A_t/A_0)$ of absorbance of Congo red at 495 nm obtained from the spectra in (a) versus time for the hydrogenation of Congo red catalyzed by the Au@CIAC-108-*heter* (in the initial 2 min).

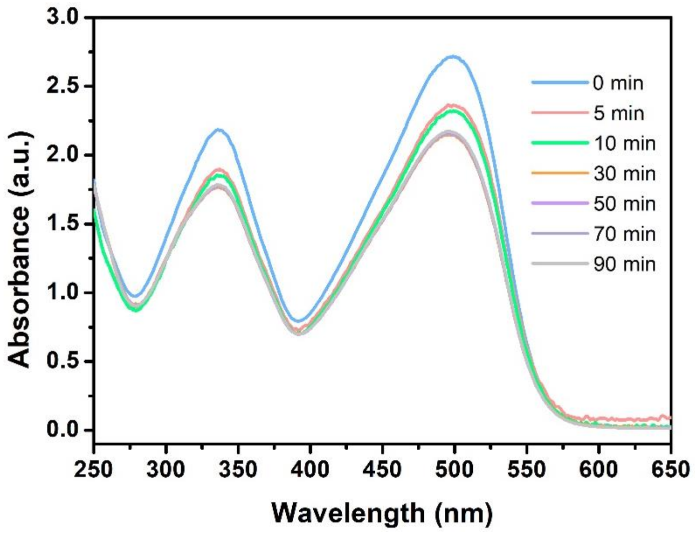

Fig. S28 UV-vis spectra of reduction of Congo red in CH_3OH/CH_2Cl_2 (v/v=1/2) in the presence of 10 mg $NaBH_4$ without catalyst. The absorption spectra show no significant changes in 90 min.

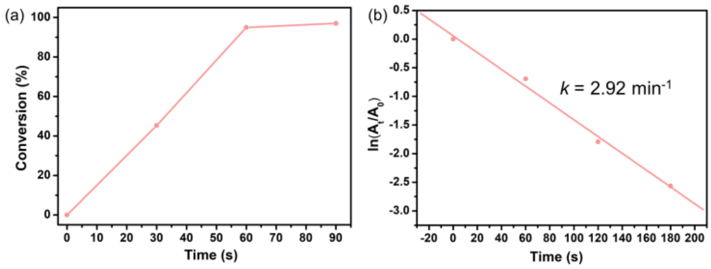

Fig. S29 (a) Catalytic conversion of methyl orange over Au@CIAC-108-*homo* in CH_3OH/CH_2Cl_2 (v/v=1/2) in the presence of 10 mg $NaBH_4$ at 298 K. (b) Plot of $\ln(A_t/A_0)$ of absorbance of methyl orange at 424 nm obtained from the spectra versus time for the hydrogenation of Congo red catalyzed by the Au@CIAC-108-*homo* catalyst.

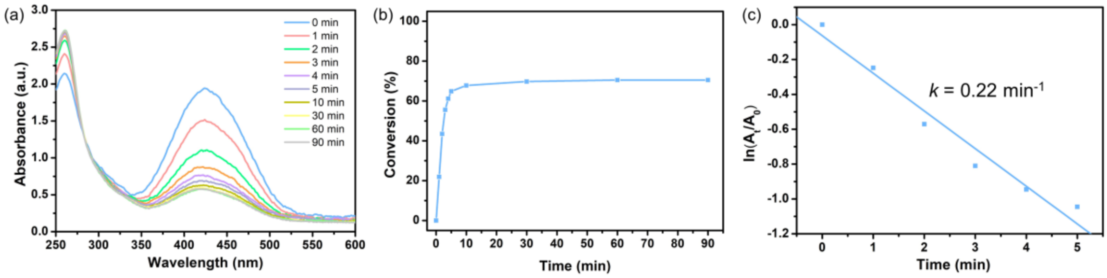

Fig. S30 (a) Catalytic conversion of methyl orange over Au/CIAC-108-*homo* in CH_3OH/CH_2Cl_2 (v/v=1/2) in the presence of 10 mg $NaBH_4$ at 298 K. (b) Plot of $\ln(A_t/A_0)$ of absorbance of methyl orange at 424 nm obtained from the spectra in (a) versus time for the hydrogenation of methyl orange catalyzed by the Au/CIAC-108-*homo* catalyst (in the initial 5 min).

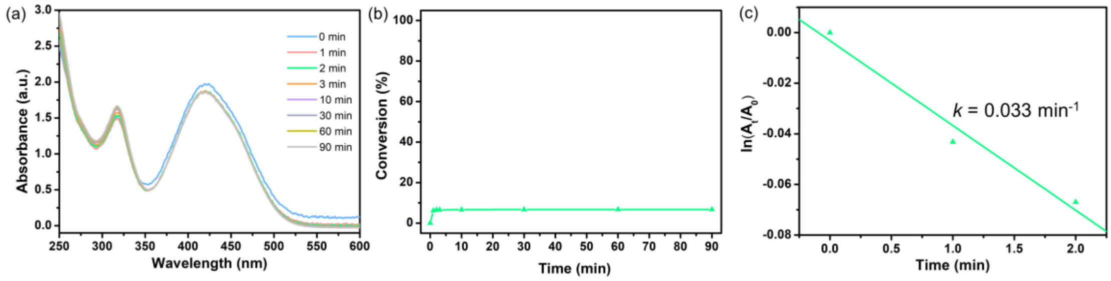

Fig. S31 (a) UV-vis spectra of reduction of methyl orange by the Au@CIAC-108-*heter* catalyst in CH_3OH in the presence of 10 mg $NaBH_4$. (b) Catalytic conversion of methyl orange over Au@CIAC-108-*heter* at 298 K. (c) Plot of $\ln(A_t/A_0)$ of absorbance of methyl orange at 424 nm obtained from the spectra in (a) versus time for the hydrogenation of methyl orange catalyzed by the Au@CIAC-108-*heter* (in the initial 2 min).

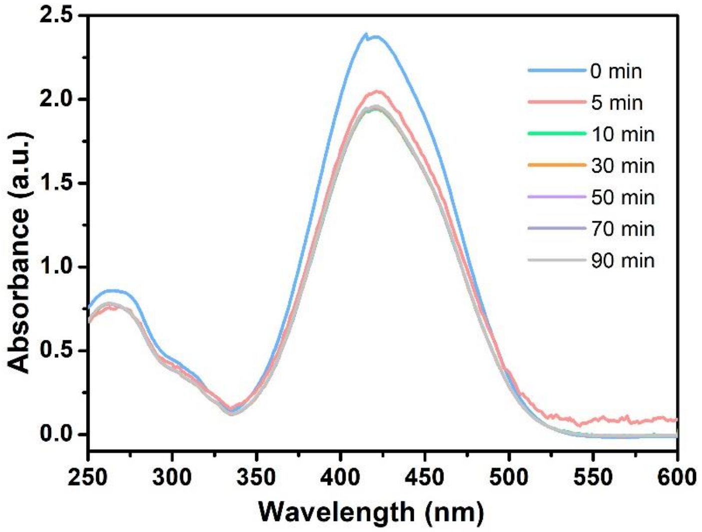

Fig. S32 UV-vis spectra of reduction of methyl orange in CH_3OH/CH_2Cl_2 (v/v=1/2) in the presence of 10 mg $NaBH_4$ without catalyst. The absorption spectra show no significant changes in 90 min.

Table S1 Comparison of rate constants of 4-nitrophenol reduction catalyzed by Au NPs catalysts

No.	Catalyst	Amount of Catalyst (mmol)	Amount of 4-NPh (mmol)	Amount of $NaBH_4$ (mmol)	Time (s)	Rate Constant k (s^{-1})	Reference
1	Au@SiO_2	1.60×10^{-3}	3.4×10^{-3}	1.20	1800	14×10^{-3}	S1
2	Au/Polyaniline	6.00×10^{-5}	3.4×10^{-4}	0.015	300	11.7×10^{-3}	S2
3	Au/Dendrimer		3.0×10^{-4}	0.03	1080	9.23×10^{-3}	S3
4	NAP-Mg-Au(0)	5.10×10^{-3}	1.08×10^{-3}	50.00	420	7.6×10^{-3}	S4
5	Au@TpPa-1	1.22×10^{-3}	2.7×10^{-3}	4.27	780	5.35×10^{-3}	S5
6	Au@Ag/ZIF-8	3.45×10^{-4}	2.7×10^{-3}	4.27	900	4.97×10^{-3}	S6
7	Au/GO	1.83×10^{-4}	7×10^{-3}	0.16	1080	3.13×10^{-3}	S7
8	Au/GO	3.73×10^{-6}	7.5×10^{-3}	2.22	1800	2.07×10^{-3}	S8
9	Au@CIAC-108	2.51×10^{-6}	5.86×10^{-4}	0.26	300	11.9×10^{-3}	This work
10	Au@PCC-1	1.65×10^{-4}	2.12×10^{-2}	0.85	96	33.7×10^{-3}	S9
11	ZIF-8 NC@Au	2.03×10^{-5}	3.85×10^{-4}	0.61	1680		S10
12	Au@MIL-100 (Fe)	4.08×10^{-2}	2.7×10^{-3}	4.27	1020	5.5×10^{-3}	S11
13	Au@TA–Fe	1.0×10^{-4}	5×10^{-4}	0.20	480	6.17×10^{-3}	S12
14	Au−HPEI10K−$IBAm_{0.80}$	2.85×10^{-5}	2.97×10^{-4}	0.03	1020		S13
15	Au-DA-polyHIPE	8.9×10^{-6}	1.2×10^{-3}	13.16	1500	6.3×10^{-3}	S14
16	Fe_3O_4@TiO_2@Au MSs		7.5×10^{-4}	0.06	180	19.67×10^{-3}	S15
17	Au@PZS@CNTs	6.40×10^{-5}	3.4×10^{-4}	0.015	960	1.78×10^{-3}	S16
18	AuNPs/SNTs	1.0×10^{-3}	3.6×10^{-3}	0.15	280	10.64×10^{-3}	S17
19	PDEAEMA–AuNPs	2.89×10^{-4}	5.0×10^{-4}	0.02	220	19.0×10^{-3}	S18
20	Au/chitosan	1.59×10^{-3}	10.0×10^{-3}	0.20	150	21.5×10^{-3}	S19
21	ICC@Au	3.0×10^{-5}	1.0×10^{-3}	13.16	300	9.33×10^{-3}	S20
22	Ni/SiO_2@Au		2.5×10^{-4}	0.20	300	10.0×10^{-3}	S21
23	KCC-1-IL/Au	2.17×10^{-6}	1.87×10^{-4}	0.25	270	12.0×10^{-3}	S22
24	Au/uTiO_2	2.52×10^{-4}	1.00×10^{-3}	0.60	360	10.5×10^{-3}	S23
25	Au/graphene hydrogel	1.22×10^{-4}	2.80×10^{-4}	0.02	720	3.17×10^{-3}	S24
26	Au/ZnO	5.00×10^{-3}	0.05	1.50	240	24×10^{-3}	S25

Table S2 Au@CIAC-108-*homo* catalyzed selective reduction of various nitroarenes[a]

entry	product	Yield[b] (%)
1	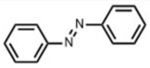	93.6
2	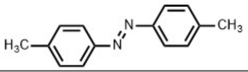	79.8
3	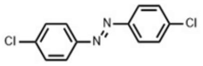	88.3

[a]Reaction conditions: different substituted nitrobenzenes (1.18 mmol), Au@CIAC-108-*homo* catalyst (0.00000753 mmol Au), NaOH (3 mmol), CH_3OH (2 mL) and CH_2Cl_2 (4 mL), room temperature, visible light irradiation (435 nm), in air. [b]Yield was determined by GC analysis (Fig. S33-35).

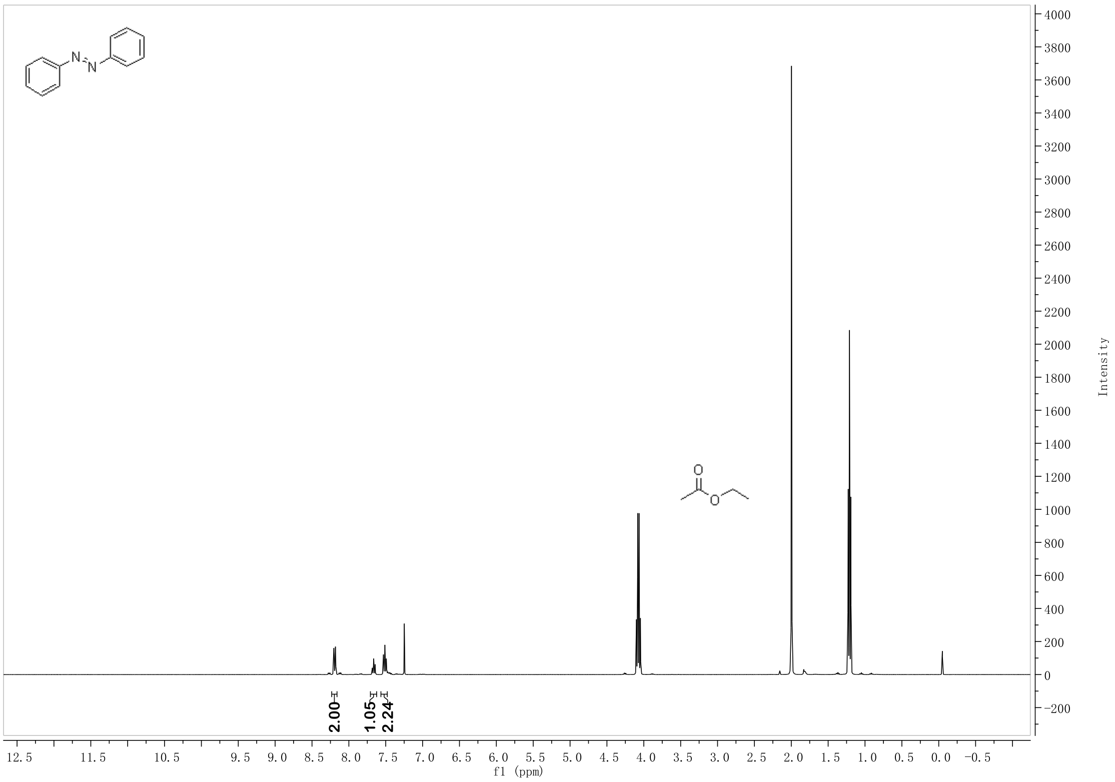

Fig. S33 ^{1}H NMR and HRMS of azobenzene: ^{1}H NMR (400 MHz, $CDCl_3$) δ 8.23–8.16 (m, 2H), 7.71–7.62 (m, 1H), 7.51 (t, *J* = 7.9 Hz, 2H). HRMS (ESI) m/z: $[M+H]^+$ calcd for $C_{12}H_{10}N_2H^+$ 183.0922; found 183.0991.

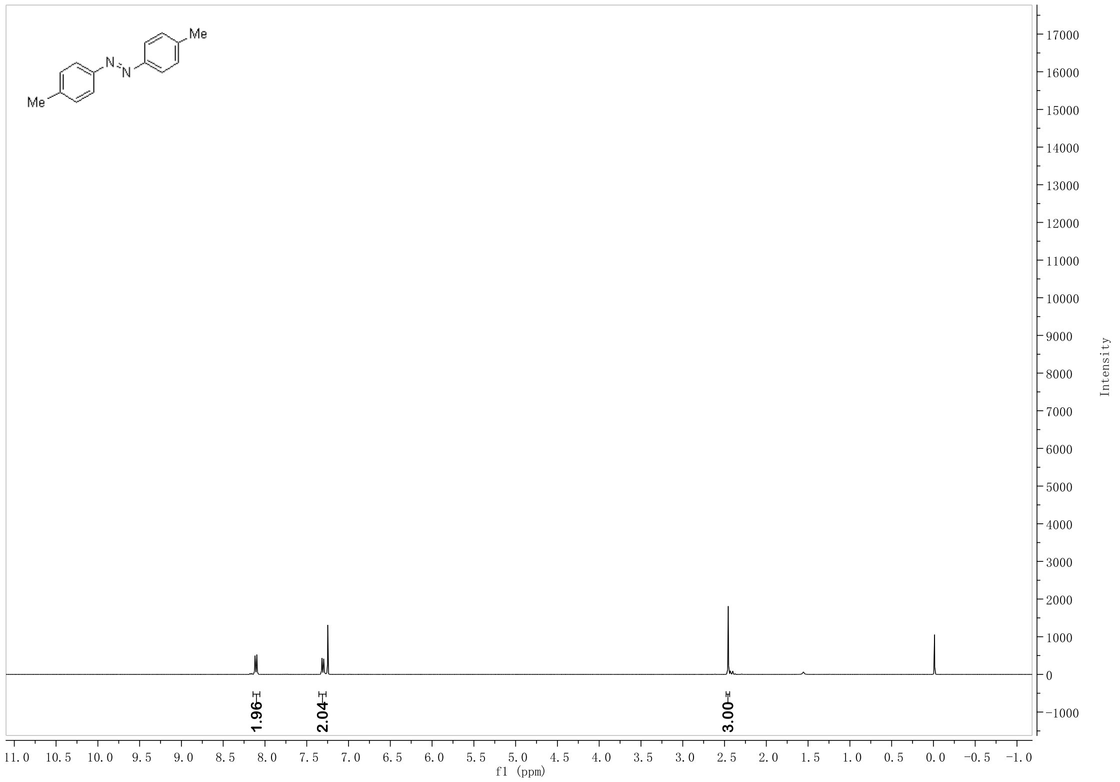

Fig. S34 ^{1}H NMR and HRMS of 4,4'-dimethylazobenzene: ^{1}H NMR (400 MHz, $CDCl_3$) δ 8.14–8.06 (m, 1H), 7.31 (dd, *J* = 8.7, 0.5 Hz, 1H), 2.46 (s, 3H). HRMS (ESI) m/z: $[M+H]^+$ calcd for $C_{14}H_{14}N_2H^+$ 211.2865; found 211.2842.

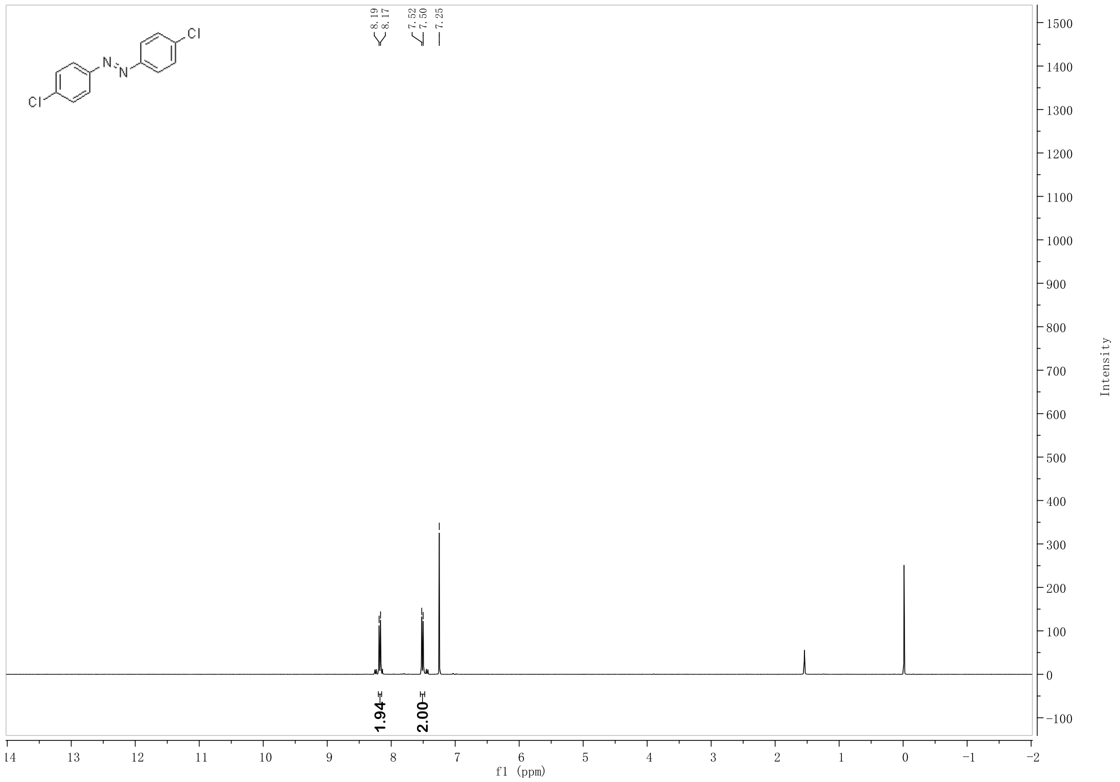

Fig. S35 ^{1}H NMR and HRMS of 4,4'-dichloroazobenzene: ^{1}H NMR (400 MHz, $CDCl_3$) δ 8.18 (d, *J* = 9.1 Hz, 2H), 7.51 (d, *J* = 9.1 Hz, 2H). HRMS (ESI) m/z: $[M+H]^+$ calcd for $C_{12}H_8Cl_2N_2H^+$ 251.1152; found 251.1147.

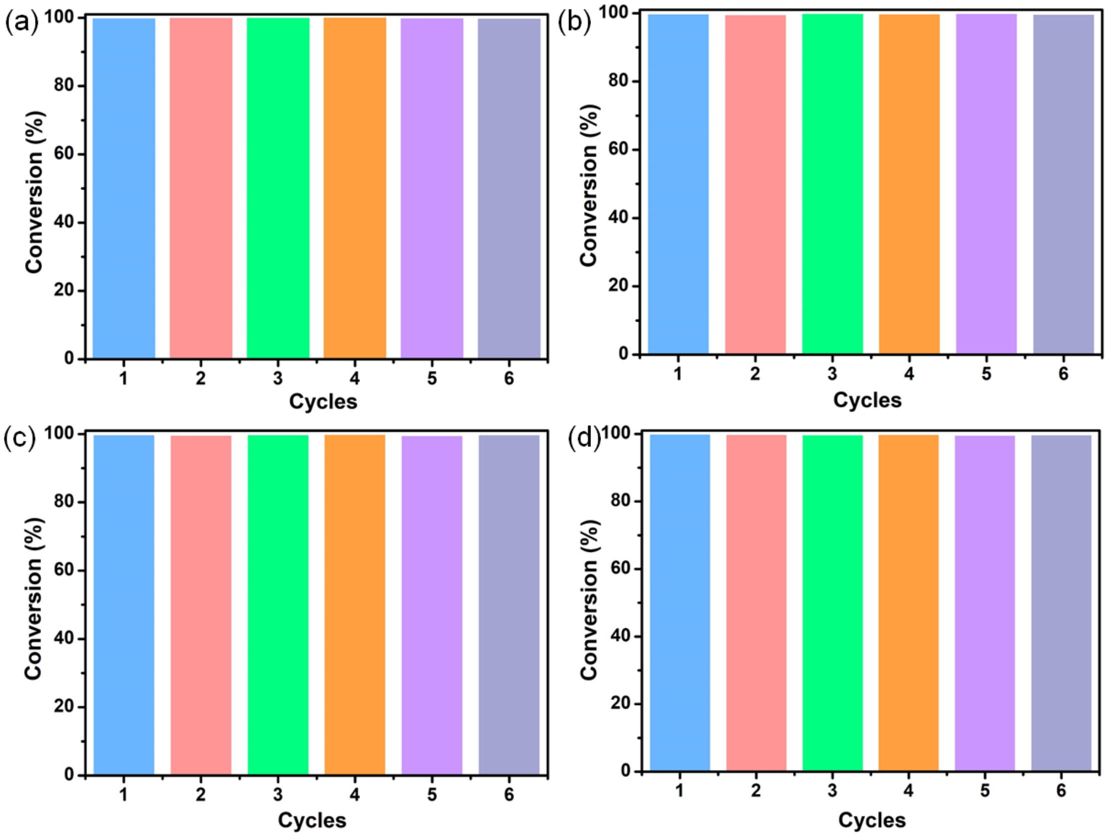

Fig. S36 Durability test for the catalytic hydrogenation of nitroarenes: (a) 2-NPh, (b) 2-NAn, (c) 3-NAn, and (d) 4-NAn over the Au@CIAC-108-*homo* catalyst.

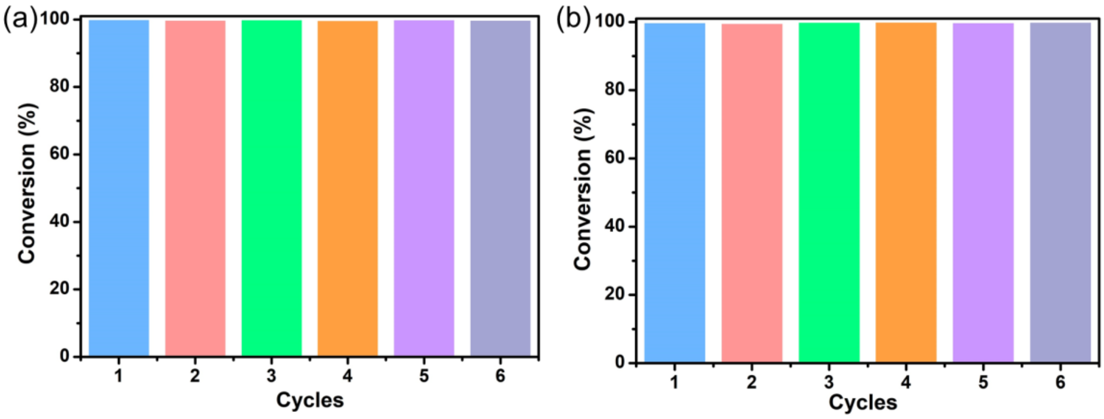

Fig. S37 Durability test for the catalytic decomposition of organic dyes: (a) Congo red and (b) methyl orange over the Au@CIAC-108-*homo* catalyst.

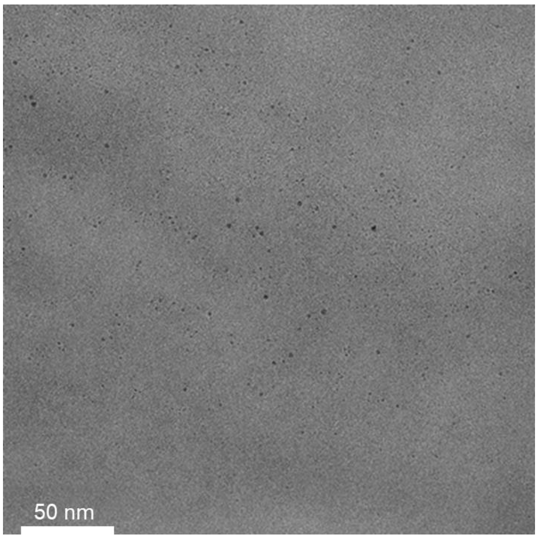

Fig. S38 TEM image of the Au@CIAC-108-*homo* catalyst after 6 cycles of the catalytic hydrogenation of 4-NPh.

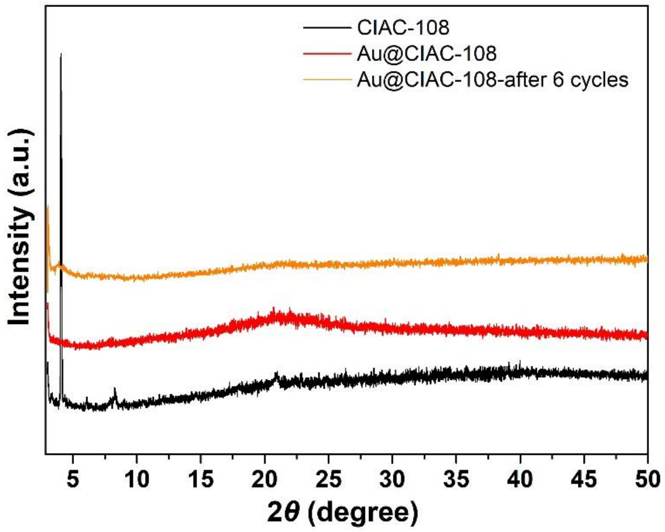

Fig. S39 PXRD pattern of Au@CIAC-108-*homo* catalyst after 6 cycles of the catalytic hydrogenation of 4-NPh.

References

S1 J. Lee, J. C. Park, H. Song, A Nanoreactor Framework of an Au@SiO_2 York/Shell Structure for Catalytic Reduction of *p*-Nitrophenol, *Adv. Mater.* **2008**, *20*, 1523-1528.

S2 J. Han, L. Y. Li, R. Cao, Novel Approach to Controllable Synthesis of Gold Nanoparticles Supported on Polyaniline Nanofibers, *Macromolecules* **2010**, *43*, 10636-10644.

S3 K. Hayakawa, T. Yoshimura, K. Esumi, Preparation of Gold-Dendrimer Nanocomposites by Laser Irradiation and Their Catalytic Reduction of 4-Nitrophenol, *Langmuir* **2003**, *19*, 5517-5521.

S4 K. Layek, M. L. Kantam, M. Shirai, D. Nishio-Hamane, T. Sasaki, H. Maheswaran, Gold Nanoparticles Stabilized on Nanocrystalline Magnesium Oxide as an Active Catalyst for Reduction of Nitroarenes in Aqueous Medium at Room Temperature, *Green Chem.* **2012**, *14*, 3164-3174.

S5 P. Pachfule, S. Kandambeth, D. D. Díaz, R. Banerjee, Highly Stable Covalent Organic Framework-Au Nanoparticles Hybrids for Enhanced Activity for Nitrophenol Reduction, *Chem. Commun.* **2014**, *50*, 3169-3172.

S6 H. L. Jiang, T. Akita, T. Ishida, M. Haruta, Q. Xu, Synergistic Catalysis of Au@Ag Core-Shell Nanoparticles Stabilized on Metal-Organic Framework, *J. Am. Chem. Soc.* **2011**, *133*, 1304-1306.

S7 Y. W. Zhang, S. Liu, W. B. Liu, L. Wang, J. Q. Tian, X. P. Sun, *In situ* Green Synthesis of Au Nanostructures on Graphene Oxide and Their Application for Catalytic Reduction of 4-Nitropheol, *Catal. Sci. Technol.* **2011**, *1*, 1142-1144.

S8 Y. Choi, H. S. Bae, E. Seo, S. Jang, K. H. Park, B. S. Kim, Hybrid Gold Nanoparticle-Reduced Graphene Oxide Nanosheets as Active Catalysts for Highly Efficient Reduction of Nitroarenes, *J. Mater. Chem.* **2011**, *21*, 15431-15436.

S9 X. X. Gou, T. Liu, Y. Y. Wang and Y. Han, Ultrastable and Highly Catalytically Active N-Heteroyclclic-Carbene-Stabilized Gold Nanoparticles in Confined Space, *Angew. Chem., Int. Ed.*, 2020, **59**, 16683-16689.

S10 Z. Li, H. C. Zeng, Surface and Bulk Integrations of Single-Layered Au or Ag Nanoparticles onto Designated Crystal Planes {110} or {100} of ZIF-8, *Chem. Mater.* **2013**, *25*, 1761-1768.

S11 F. Ke, J. F. Zhu, L. G. Qiu, X. Jiang, Controlled Synthesis of Novel Au@MIL-100(Fe) Core-Shell Nanoparticles with Enhanced Catalytic Performance, *Chem. Commun.* **2013**, *49*, 1267-1269.

S12 T. Zeng, X. L. Zhang, Y. Y. Guo, H. Y. Niu, Y. Q. Cai, Enhanced Catalytic Application of Au@Polyphenol-Metal Nanocomposites Synthesized by A Facile and Green Method, *J. Mater. Chem. A* **2014**, *2*, 14807-14811.

S13 Y. Liu, L. Xu, X. Y. Liu, M. N. Cao, Hybrids of Gold Nanoparticles with Core-Shell Hyperbranched Polymers: Synthesis, Characterization, and Their High Catalytic Activity for Reduction of 4-Nitrophenol, *Catalysts* **2016**, *6*, 3-16.

S14 Y. L. Ye, M. Jin, D. C. Wan, One-Pot Synthesis of Porous Monolith-Supported Gold Nanoparticles as an Effective Recyclable Catalyst, *J. Mater. Chem. A* **2015**, *3*, 13519-13525.

S15 Y. Zhou, Y. H. Zhu, X. L. Yang, J. F. Huang, W. Chen, X. M. Lv, C. Y. Li, C. Z. Li, Au Decorated Fe_3O_4@TiO_2 Magnetic Composites with Visible Light-Assisted Enhanced Catalytic Reduction of 4-Nitrophenol, *RSC Adv.* **2015**, *5*, 50454-50461.

S16 X. Z. Wang, J. W. Fu, M. H. Wang, Y. J. Wang, Z. M. Chen, J. N. Zhang, J. F. Chen, Q. Xu, Facile Synthesis of Au Nanoparticles Supported on Polyphosphazene Functionalized Carbon Nanotubes for Catalytic Reduction of 4-Nitrophenol, *J Mater Sci* **2014**, *49*, 5056-5065.

S17 Z. Y. Zhang, C. L. Shao, P. Zou, P. Zhang, M.Y. Zhang, J. B. Mu, Z. C. Guo, X. H. Li, C. H. Wang, Y. C. Liu, *In situ* Assembly of Well-Dispersed Gold Nanoparticles on Electrospum Silica Nanotubes for Catalytic Reduction of 4-Nitrophenol, *Chem. Commun.* **2011**, *47*, 3906-3908.

S18 J. M. Zhang, D. H. Han, H. J. Zhang, M. Chaker, Y. Zhao, D. L. Ma, *In situ* Recyclable Gold Nanoparticles Using CO_2-Switchable Polymers for Catalytic Reduction of 4-Nitrophenol, *Chem. Commun.* **2012**, *48*, 11510-11512.

S19 Y. F. Qiu, Z. Ma, P. A. Hu, Environmentally Benign Magnetic Chitosan/Fe_3O_4 Composites as Reductant and Stabilizer for Anchoring Au NPs and Their Catalytic Reduction of 4-Nitrophenol, *J. Mater. Chem. A* **2014**, *2*, 13471-13478.

S20 R. Xiong, Y. R. Wang, X. X. Zhang, C. H. Liu, L. D. Lan, In situ Growth of Gold Nanoparticles on Magnetic γ-Fe_2O_3@Cellulose Nanocomposites: A Highly Active and Recyclable Catalyst for Reduction of 4-Nitrophenol, *RSC Adv.* **2014**, *4*, 6454-6462.

S21 S. H. Zhang, S. L. Gai, F. He, Y. L. Dai, P. Gao, L. Li, Y. J. Chen, P. P. Yang, Uniform Ni/SiO_2@Au Magnetic Hollow Microspheres: Rational Design and Excellent Catalytic Performance in 4-Nitrophenol Reduction, *Nanoscale* **2014**, *6*, 7025-7032.

S22 H. L. Yang, S. W. Li, X. Y. Zhang, X. Y. Wang, J. T. Ma, Imidazolium Ionic Liquid-Modified Fibrous Silica Microspheres Loaded with Gold Nanoparticles and Their Enhanced Catalytic Activity and Reusability for the Reduction of 4-Nitrophenol, *J. Mater. Chem. A* **2014**, *2*, 12060-12067.

S23 Z. H. Ren, H. T. Li, Q. Gao, H. Wang, B. Han, K. S. Xia, C. G. Zhou, Au Nanoparticles Embedded on Urchin-like TiO_2 Nanosphere: An Efficient Catalyst for Dyes degradation and 4-Nitrophenol Reduction, *Mater. Design* **2017**, *121*, 167-175.

S24 J. Li, C. Y. Liu, Y. Liu, Au/Graphene Hydrogel: Synthesis, Characterization and Its Use for Catalytic Reduction of 4-Nitrophenol, *J. Mater. Chem.* **2012**, *22*, 8426-8430.

S25 H. Koga, T. Kitaoka, One-step Synthesis of Gold Nanocatalysts on a Microstructured Paper Matrix for the Reduction of 4-Nitrophenol, *Chem. Eng. J.* **2011**, *168*, 420-425.
